# Au–Pd alloy nanoparticles supported on layered double hydroxide for heterogeneously catalyzed aerobic oxidative dehydrogenation of cyclohexanols and cyclohexanones to phenols†
†Electronic supplementary information (ESI) available. See DOI: 10.1039/c6sc00874g


**DOI:** 10.1039/c6sc00874g

**Published:** 2016-05-06

**Authors:** Xiongjie Jin, Kento Taniguchi, Kazuya Yamaguchi, Noritaka Mizuno

**Affiliations:** a Department of Applied Chemistry , School of Engineering , The University of Tokyo , 7-3-1 Hongo, Bunkyo-ku , Tokyo 113-8656 , Japan . Email: tmizuno@mail.ecc.u-tokyo.ac.jp ; Fax: +81-3-5841-7220

## Abstract

Phenol, an important industrial chemical, is widely produced using the well-developed cumene process. However, demand for the development of a novel alternative method for synthesizing phenol from benzene has been increasing. Herein, we report a novel system for the synthesis of phenols through aerobic oxidative dehydrogenation of cyclohexanols and cyclohexanones, including ketone–alcohol (KA) oil, catalyzed by Mg–Al-layered double hydroxide (LDH)-supported Au–Pd alloy nanoparticles (Au–Pd/LDH). Alloying of Au and Pd and basicity of LDH are key factors in achieving the present transformation. Although monometallic Au/LDH, Pd/LDH, and their physical mixture showed almost no catalytic activity, Au–Pd/LDH exhibited markedly high catalytic activity for the dehydrogenative phenol production. Mechanistic studies showed that β-H elimination from Pd-enolate species is accelerated by Au species, likely *via* electronic ligand effects. Moreover, the effect of supports was critical; despite the high catalytic performance of Au–Pd/LDH, Au–Pd bimetallic nanoparticles supported on Al_2_O_3_, TiO_2_, MgO, and CeO_2_ were ineffective. Thus, the basicity of LDH plays a deterministic role in the present dehydrogenation possibly through its assistance in the deprotonation steps. The synthetic scope of the Au–Pd/LDH-catalyzed system was very broad; various substituted cyclohexanols and cyclohexanones were efficiently converted into the corresponding phenols, and *N*-substituted anilines were synthesized from cyclohexanones and amines. In addition, the observed catalysis was truly heterogeneous, and Au–Pd/LDH could be reused without substantial loss of its high performance. The present transformation is scalable, utilizes O_2_ in air as the terminal oxidant, and generates water as the only by-product, highlighting the potential practical utility and environmentally benign nature of the present transformation. Dehydrogenative aromatization of cyclohexanols proceeds through (1) oxidation of cyclohexanols to cyclohexanones; (2) dehydrogenation of cyclohexanones to cyclohexenones; and (3) disproportionation of cyclohexenones to afford the desired phenols. In the present Au–Pd/LDH-catalyzed transformation, the oxidation of the Pd–H species is included in the rate-determining step.

## Abstract 2

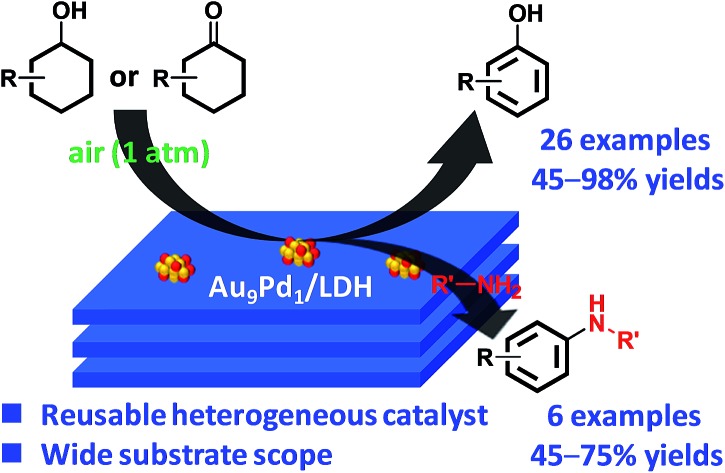
We developed a novel system for the synthesis of phenols through aerobic oxidative dehydrogenation of cyclohexanols and cyclohexanones by Mg–Al-layered double hydroxide-supported Au–Pd alloy nanoparticles.

## Introduction

**Scheme 1:**
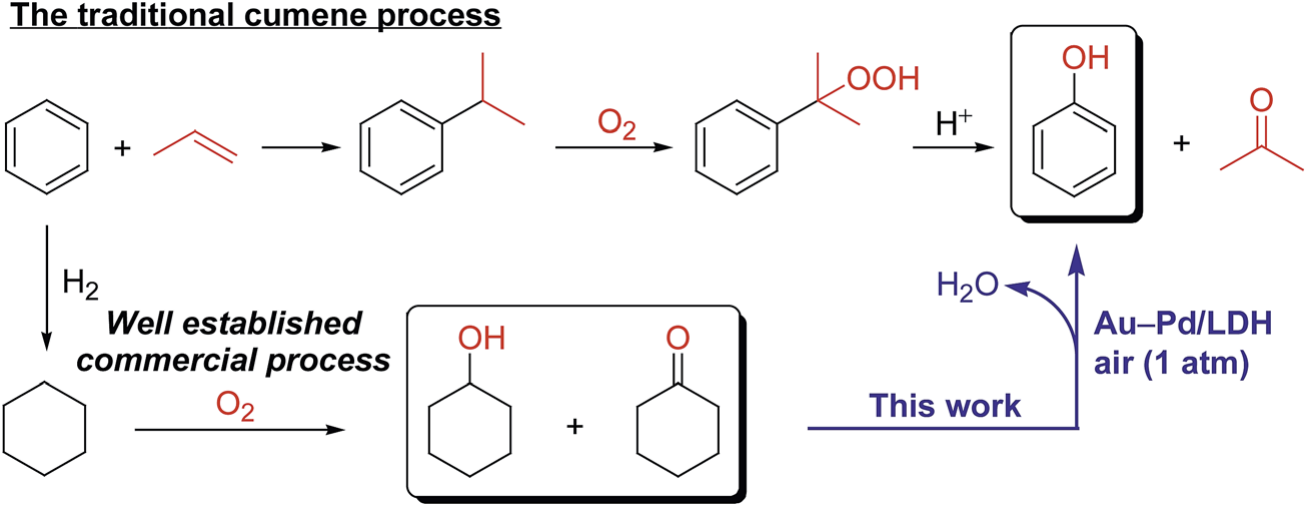
Synthetic procedures for phenol.

Phenol and its derivatives are very important industrial chemicals and key structural motifs in various pharmaceuticals, agrochemicals, plastics, and resins.^1^ Annually, millions of tons of phenol are produced mainly by the well-established three-step cumene process starting from benzene (Scheme 1).^2^ However, this traditional cumene process suffers from several drawbacks, including the formation of acetone co-product and the involvement of an explosive cumene hydroperoxide intermediate.^2^ Although the current cumene process is the most competitive and widely employed commercial process for phenol, the development of new processes that avoid these problems is highly desirable. In this context, processes for the one-step direct oxygenation of benzene to phenol using various oxidants such as H_2_O_2_, N_2_O, and O_2_ have attracted considerable attention as promising alternatives for producing phenols.^3^ However, little success has been achieved in the field of direct selective hydroxylation of benzene to phenol, largely because of the inertness of C–H bonds in benzene and the more facile oxidation of phenol than benzene by oxidizing agents, leading to overoxidation.^3^ Dehydrogenative aromatization of a mixture of cyclohexanone and cyclohexanol—specifically, an unrefined mixture of cyclohexanone and cyclohexanol, known as ketone–alcohol (KA) oil—can provide an alternative approach to access phenol from benzene. KA oil is readily available through a well-developed two-step commercial process involving the hydrogenation of benzene to cyclohexane and aerobic autoxidation of cyclohexane (Scheme 1).

Recently, several homogeneous Pd-based catalytic systems for the dehydrogenation of cyclohexanones to phenols using O_2_ as the terminal oxidant under mild reaction conditions have been developed.^4^ These systems are highly valuable for the synthesis of phenols with various substitution patterns given (1) the limitations of traditional electrophilic substitution reactions for the regioselective introduction of substituents onto a phenol ring as a consequence of the strong electron-directing effects of hydroxy groups and (2) the easy accessibility of cyclohexanones with various substituents on cyclohexyl rings through a wide range of chemical transformations. Acceptorless dehydrogenation of cyclohexanones using a homogeneous Ir-based catalyst has also been developed.^5^ Despite the high efficiency of these homogeneous systems, they suffer several drawbacks, including the requirement of rather sophisticated ligands and/or high reaction temperatures and difficulties associated with the separation and reuse of the catalysts. Quite recently, a supported Pd-nanoparticle-catalyzed dehydrogenation of cyclohexanones has also been developed either using O_2_ (5 atm) as a hydrogen acceptor6*a* or under acceptorless conditions.6*b* However, in these cases, the need for large amounts of bases as additives and/or leaching of Pd species is problematic in terms of catalyst stability and reusability. Therefore, the development of novel catalytic systems using easily separable and reusable heterogeneous catalysts under ligand- and additive-free conditions is highly desirable from practical, economical, and environmental viewpoints. Despite remarkable progress in the dehydrogenation of cyclohexanones to phenols in recent years, as far as we know, the literature contains no reports of efficient catalytic systems that directly convert cyclohexanols to phenols with useful yields under relatively mild conditions. Therefore, the development of novel catalytic systems that facilitate dehydrogenation of cyclohexanols to phenols would not only enable the synthesis of phenol directly using KA oil as the feedstock without tedious isolation of cyclohexanone, but would also complement the aforementioned synthesis of substituted phenols *via* the dehydrogenation of cyclohexanones.

Over the past several decades, (supported) metal nanoparticles have attracted much attention as efficient catalysts for a wide variety of aerobic oxidation reactions.^7^ In particular, multi-component noble-metal nanoparticle catalysts have often shown catalytic activities superior to those of their monometallic counterparts because of the synergistic effects between different components.^8^ In recent years, Au-based multi-metallic nanoparticles have been among the most extensively studied catalysts.^9^ For example, Au–Pd bimetallic nanoparticle catalysts have shown significantly higher catalytic activity than Au or Pd monometallic nanoparticles catalysts for oxygen-related transformations such as the aerobic oxidations of CO to CO_2_,^10^ alcohols to carbonyl compounds,^11^ and H_2_ to H_2_O_2_.^12^ The high catalytic activity of multi-metallic nanoparticles is attributable to the ligand and ensemble effects, which can affect the local electronic and geometric structures of nanoparticles, thereby leading to enhancement of the catalytic performance.^8^ In the case of supported multi-metallic nanoparticles, in addition to their structures (*e.g.*, core–shell, hetero, or alloyed structures), the choice of appropriate supports is also key to achieving high catalytic performance. The support can not only stabilize the highly dispersed metal nanoparticles, but also substantially promote the reaction through metal-support cooperation.^13^ Therefore, we envisage that Pd nanoparticles alloyed with other metals (*e.g.*, Au) supported on suitable support materials could promote the aerobic oxidative dehydrogenation of cyclohexanols and cyclohexanones to phenols.

Herein, we report for the first time that Au–Pd bimetallic alloy nanoparticles supported on Mg–Al-layered double hydroxide^14^ (Au–Pd/LDH) can efficiently promote the aerobic oxidative dehydrogenation of various structurally diverse cyclohexanols to phenols *via* a consecutive triple-dehydrogenation process. Interestingly, whereas the bimetallic Au–Pd/LDH exhibits high catalytic performance, monometallic Au/LDH and Pd/LDH and their physical mixture exhibit almost no catalytic activity toward the dehydrogenation. The catalysis for the present dehydrogenative aromatization was truly heterogeneous, and the Au–Pd/LDH catalyst could be reused at least 10 times with retention of its high catalytic performance.

## Results and discussion

### Preparation and characterization of LDH-supported Au–Pd alloy nanoparticles

Initially, we prepared several Au–Pd/LDH catalysts with different Au/Pd ratios using the deposition–precipitation method. Specifically, Au and Pd species were co-precipitated onto the LDH surface as hydroxide species and these metal species were subsequently reduced using NaBH_4_ (for details on the preparation procedures for the Au–Pd/LDH catalysts, see the Experimental section). As revealed by inductively coupled plasma atomic emission spectroscopy (ICP-AES) analysis, the Au/Pd molar ratios of the Au–Pd/LDH catalysts slightly deviated from those of the initial metal precursors used to prepare the catalysts. Hereafter, Au–Pd/LDH catalysts with an initial solution Au/Pd molar ratio of *x*/*y* are designated as Au_*x*_Pd_*y*_/LDH. The metal contents of Au_*x*_Pd_*y*_/LDH are summarized in Table S1.†

**Fig. 1:**
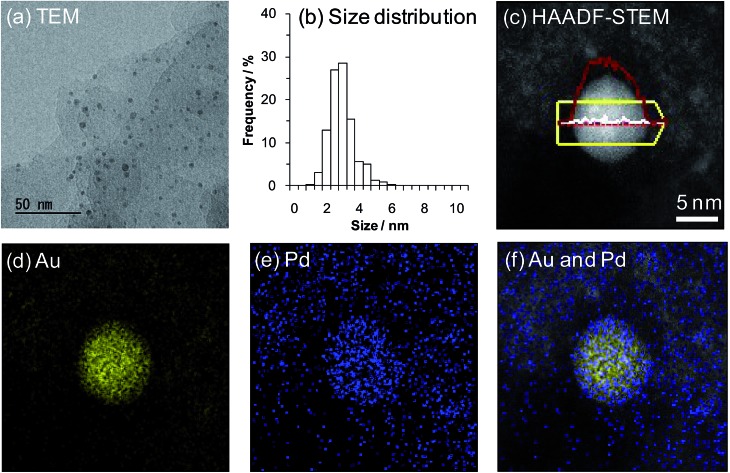
(a) TEM image of Au_9_Pd_1_/LDH. (b) The size distribution of Au_9_Pd_1_/LDH. (c) HAADF-STEM image of Au_9_Pd_1_/LDH. EDS intensity line profiles are shown in red (Au) and white (Pd) lines. (d) EDS elemental map of Au. (e) EDS elemental map of Pd. (f) Overlap of EDS elemental map of Au and Pd.

X-ray diffraction (XRD) patterns of the catalysts showed that the layered structure of LDH was well maintained after immobilization of the Au–Pd bimetallic nanoparticles onto the surface (Fig. S1†). Transmission electron microscopy (TEM) analysis revealed that the average Au–Pd bimetallic nanoparticle size of Au_9_Pd_1_/LDH was 3.2 nm (Fig. 1a and b; for the average metal nanoparticle sizes of other catalysts, see Table S1†). In addition, high-angle annular dark-field scanning TEM (HAADF-STEM) and energy dispersive X-ray spectroscopy (EDS) analyses of Au_9_Pd_1_/LDH indicated that Au–Pd alloy nanoparticles were formed on the surface of LDH.^15^ This result was also supported by the UV-vis spectrum of Au_9_Pd_1_/LDH; the intensity of the absorption band at approximately 520 nm, which is derived from plasmon resonance absorption of Au nanoparticles, was markedly decreased compared to that of monometallic Au/LDH (Fig. S2†).^16^ Similarly, UV-vis spectra of other supported bimetallic nanoparticle catalysts Au_*x*_Pd_*y*_/LDH also indicated the formation of Au–Pd alloy nanoparticles (Fig. S2†).

### Optimization of reaction conditions

Next, various supported Au–Pd alloy nanoparticles were applied to the dehydrogenative aromatization of 4-methylcyclohexanol (**1a**) to 4-methylphenol (**2a**) (Table 1). The reaction was carried out in *N*,*N*-dimethylacetamide (DMA) at 130 °C under open-air conditions. Although monometallic Au/LDH and Pd/LDH promoted the oxidation of **1a** to 4-methylcyclohexanone (**3a**), the desired **2a** through the dehydrogenative aromatization of **3a** was not obtained in either case (Table 1, entries 1 and 6). Surprisingly, when the reaction was carried out with Au_9_Pd_1_/LDH as the catalyst, 91% yield of **2a** was obtained (Table 1, entry 2). The effect of the Au/Pd molar ratio on the present transformation was significant. The catalytic activity decreased with decreasing Au/Pd molar ratio (Table 1, entries 1–6), and Au_1_Pd_1_/LDH and Au_1_Pd_4_/LDH hardly gave **2a** (Table 1, entries 4 and 5). The effect of the support on the present reaction was also critical. Au–Pd bimetallic nanoparticles supported on other materials such as Al_2_O_3_, TiO_2_, and CeO_2_ did not exhibit catalytic activity for the dehydrogenation; in these cases, neither **2a** nor **3a** was formed (Table 1, entries 7–9). Although the dehydrogenation of **1a** gave **3a** when using MgO as the support for Au–Pd alloy nanoparticles, **2a** was not formed at all (Table 1, entry 10). Notably, the aerobic oxidation of **1a** to **3a** by Au_4_Pd_1_/Al_2_O_3_ was significantly promoted by addition of bases such as K_2_CO_3_ (stoichiometric amount, 0.5 mmol) and LDH (100 mg) to the reaction mixture (Table 1, entries 11–13). Thus, the basicity of the LDH support likely plays a key role in promoting the oxidation of **1a** to **3a** through its assistance of the abstraction of the proton from the hydroxy group of **1a**.^14^ The dehydrogenative aromatization of **1a** with a physical mixture of Au/LDH and Pd/LDH (where the Au/Pd ratio was the same as that of Au_9_Pd_1_/LDH) only gave a trace amount of **2a** and a moderate yield of **3a** (Table 1, entry 14), which indicates that formation of the bimetallic alloy is indispensable for the effective transformation of **3a** to **2a**. In addition, the reaction with a physical mixture of Au_4_Pd_1_/Al_2_O_3_ or Au_9_Pd_1_/TiO_2_ and LDH did not give **2a** at all, thus suggesting that highly dispersed Au–Pd bimetallic alloy nanoparticles on the surface of LDH are the key to achieving highly efficient dehydrogenative aromatization of **3a** to **2a** (Table 1, entries 13 and 15). The reaction under an Ar atmosphere gave only a trace amount of **3a** and did not give **2a**, which suggests that O_2_ in air functioned as the terminal oxidant in the present system (Table 1, entry 16). In the absence of a catalyst, the reaction did not proceed (Table 1, entry 17). Among the various solvents examined, *i.e.*, DMA, *N*,*N*-dimethylformamide (DMF), *N*-methylpyrrolidone (NMP), dimethylsulfoxide (DMSO), monochlorobenzene, and mesitylene, DMA best promoted the present dehydrogenation (Table S2†).^17^

**Table 1:** Oxidative dehydrogenation of 4-methylcyclohexanol (**1a**) to 4-methylphenol (**2a**) with various catalysts^*a*^

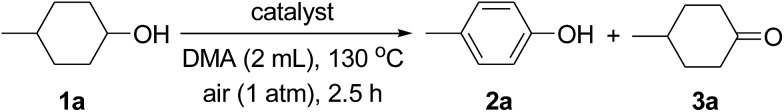					
Entry	Catalyst	Conv. (%)	Yield (%)		Au/Pd^*b*^
			**2a**	**3a**	
1	Au/LDH	55	nd	55	3.6/0
**2**	**Au**_**9**_ **Pd**_**1**_ **/LDH**	**94**	**91**	**2**	**3.1/0.5**
3	Au_4_Pd_1_/LDH	87	79	4	2.6/1.0
4	Au_1_Pd_1_/LDH	52	2	29	1.5/2.1
5	Au_1_Pd_4_/LDH	59	nd	43	0.7/2.9
6	Pd/LDH	56	nd	45	0/3.6
7	Au_9_Pd_1_/Al_2_O_3_	<1	nd	nd	3.2/0.4
8	Au_9_Pd_1_/TiO_2_	<1	nd	nd	3.1/0.5
9	Au_9_Pd_1_/CeO_2_	<1	nd	nd	3.2/0.4
10	Au_9_Pd_1_/MgO	31	nd	21	2.9/0.7
11	Au_4_Pd_1_/Al_2_O_3_	17	nd	11	2.8/0.8
12^*c*^	Au_4_Pd_1_/Al_2_O_3_ + K_2_CO_3_	60	nd	35	2.8/0.8
13^*d*^	Au_4_Pd_1_/Al_2_O_3_ + LDH	32	nd	22	2.8/0.8
14^*e*^	Au/LDH + Pd/LDH	73	5	53	3.1/0.5
15^*d*^	Au_9_Pd_1_/TiO_2_ + LDH	2	nd	2	3.1/0.5
16^*f*^	Au_9_Pd_1_/LDH	3	nd	3	3.1/0.5
17	None	21	nd	nd	—

^*a*^Reaction conditions: **1a** (0.5 mmol), catalyst (total amount of metals: 3.6 mol%), DMA (2 mL), 130 °C, air (1 atm), 2.5 h. Conversion and yields were determined by GC analysis. DMA = *N*,*N*-dimethylacetamide.

^*b*^Amount of metals (mol%).

^*c*^K_2_CO_3_ (0.5 mmol).

^*d*^LDH (100 mg).

^*e*^A physical mixture of Au/LDH (3.1 mol%) and Pd/LDH (0.5 mol%).

^*f*^Ar (1 atm).

The conditions for the reaction starting from **3a** were also optimized. As shown in Table S3,† Au/LDH and Pd/LDH exhibited almost no catalytic activity toward the dehydrogenation of **3a** (Table S3,† entries 1 and 6). For the reaction of **3a**, Au_9_Pd_1_/LDH also exhibited the best performance among various LDH-supported Au–Pd alloy nanoparticle catalysts (Table S3,† entries 2–5). Au–Pd alloy nanoparticles supported on Al_2_O_3_, TiO_2_, CeO_2_, and MgO gave only trace amounts of **2a** (Table S3,† entries 7–10). The dehydrogenation of **3a** using Au_4_Pd_1_/Al_2_O_3_ as the catalyst was substantially promoted by the addition of K_2_CO_3_ or LDH (Table S3,† entries 11–13). These results strongly support the hypothesis that the basicity of LDH plays the key role in the dehydrogenative aromatization of **3a**, likely *via* its assistance in the abstraction of α-H from **3a**.^14^ The physical mixture of Au/LDH and Pd/LDH did not exhibit catalytic activity for the dehydrogenative aromatization (Table S3,† entry 14), and the physical mixture of Au_9_Pd_1_/TiO_2_ and LDH afforded a lower yield of **2a** than Au_9_Pd_1_/LDH (Table S3,† entry 15), indicating the highly dispersed Au–Pd alloy nanoparticles on the surface of LDH are indispensable for the dehydrogenative aromatization of **3a**. The transformation of **3a** under an Ar atmosphere did not give **2a**, which suggests that O_2_ in air is the terminal oxidant (Table S3,† entry 16). These results related to the transformation of **3a** agree well with those obtained starting from **1a**.

### Heterogeneous catalysis and reusability of Au_9_Pd_1_/LDH

**Fig. 2:**
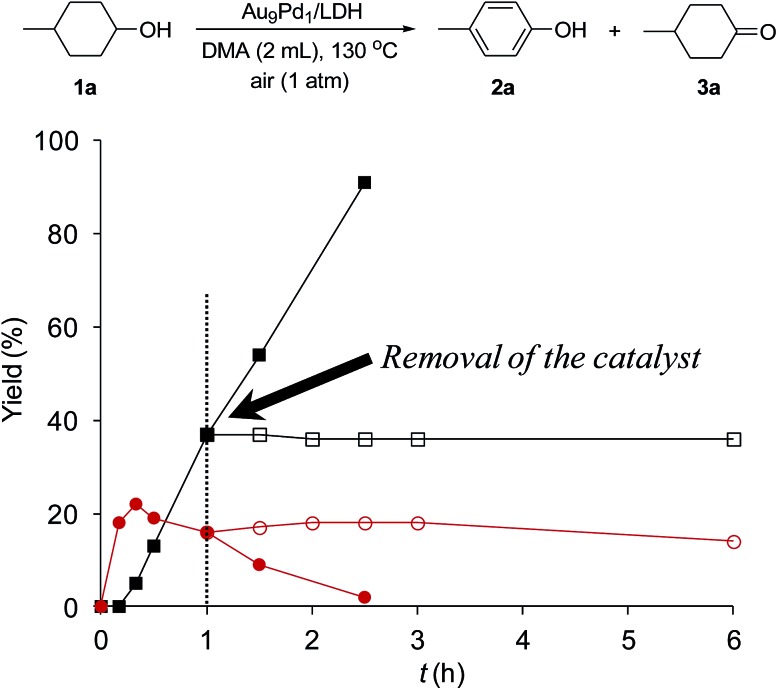
Effect of removal of the Au_9_Pd_1_/LDH catalyst on the present oxidative dehydrogenation. The reaction conditions were the same as those described in Table 1. GC yields are shown here. Closed squares and circles indicate the yields of **2a** and **3a**, respectively, without removal of the catalyst; open squares and circles indicate the yields of **2a** and **3a**, respectively, after removal of the catalyst.

To verify whether the observed catalysis was truly heterogeneous, the Au_9_Pd_1_/LDH catalyst was removed by hot filtration when the conversion of **1a** reached approximately 40%, and the reaction was repeated using the filtrate under the same reaction conditions. As shown in Fig. 2, the reaction was completely stopped by the removal of the catalyst. Furthermore, the ICP-AES analysis results confirmed that Au and Pd species were hardly present in the filtrate (below the instrumental detection limits: Au < 0.01%, Pd < 0.38%). On the basis of these experimental results, we ruled out the possibility that the catalysis originated from metal species leached from the catalyst; thus, the observed catalysis was truly heterogeneous.^18^

**Fig. 3:**
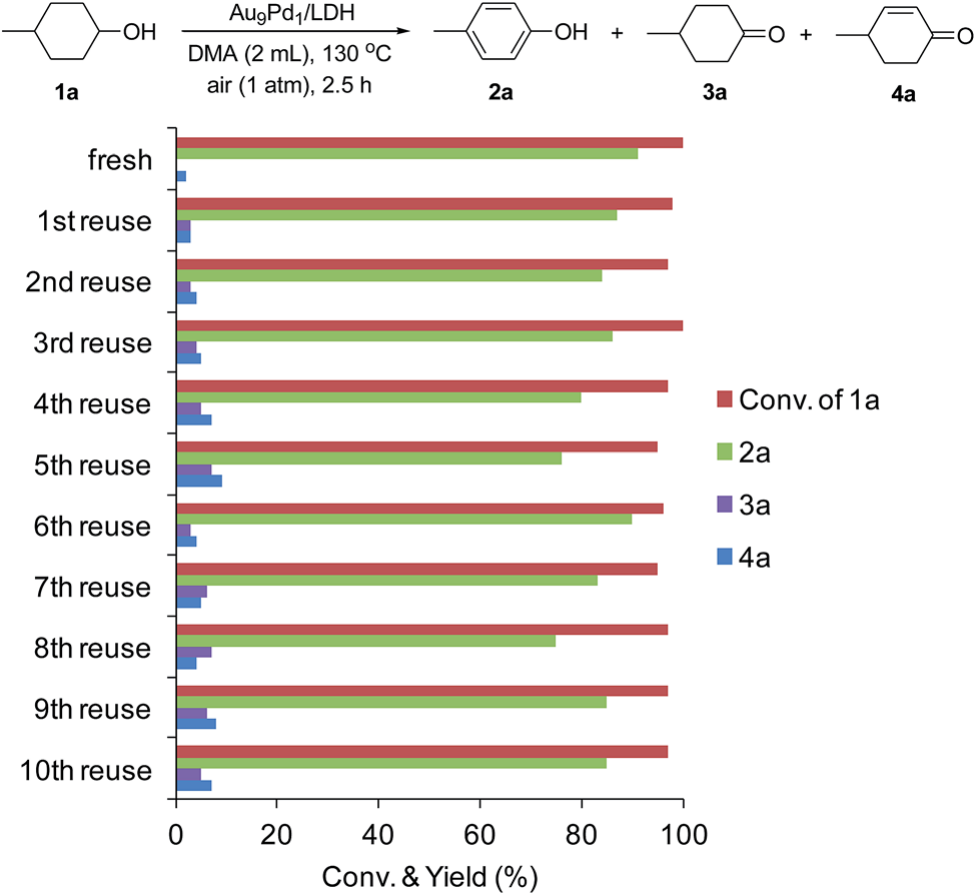
Au_9_Pd_1_/LDH reuse experiments. The reaction conditions were the same as those described in Table 1. GC yields are shown here. Generally, the retrieved catalyst was washed with water and ethanol. However, the retrieved catalysts after 5th, 8th, and 9th reuse experiments were washed with a 0.1 M NaOH solution in water/EtOH (1 : 1).

After the dehydrogenation of **1a** to **2a** was completed, Au_9_Pd_1_/LDH was easily retrieved from the reaction mixture by simple filtration, with greater than 95% recovery, for each reuse experiment. The retrieved catalyst could be reused at least 10 times without substantial loss of its high catalytic performance. Even at the 10th reuse experiment, 85% yield of **2a** was still obtained (Fig. 3). The catalytic performance could be restored by simply washing the used catalyst with a 0.1 M NaOH solution in water/ethanol (1 : 1). XRD and TEM analyses of the fresh as-prepared Au_9_Pd_1_/LDH catalyst and the catalyst after the 10th reuse experiment show that the LDH structure and the average size of the Au–Pd bimetallic alloy nanoparticles were almost unchanged (fresh: 3.2 nm, after the 10th reuse experiment: 3.3 nm) (Fig. S3 and S4†). The difference UV-vis spectrum between fresh Au_9_Pd_1_/LDH and Au_9_Pd_1_/LDH after the 10th reuse experiment revealed a new absorption band centered at approximately 250 nm, which is attributable to aromatic compounds (possibly **2a**) (Fig. S5†). These results indicate that the gradual decrease of the catalytic activity is likely caused by the absorption of acidic phenol onto the surface of the catalyst. Thus, the catalytic ability (basicity) of the LDH support was readily recovered by washing with the NaOH solution, thereby restoring the catalytic activity of Au_9_Pd_1_/LDH.

### Substrate scope

With the optimized reaction conditions in hand, we next investigated the scope of the present Au_9_Pd_1_/LDH-catalyzed dehydrogenative aromatization of cyclohexanols. Under the optimized reaction conditions, various structurally diverse cyclohexanols were efficiently converted into the corresponding phenols. The phenol products were readily isolated; their yields are summarized in Table 2. The dehydrogenation of simple cyclohexanol afforded phenol in 89% yield (Table 2, entry 1). The dehydrogenation of cyclohexanols with various substituents at 2-, 3-, and 4-positions smoothly proceeded (Table 2, entries 2–12). 2-Substituted cyclohexanols were not as reactive as 3- and 4-substituted ones, suggesting that steric effects on the dehydrogenation are substantial (Table 2, entries 2, 3, and 8). Various disubstituted cyclohexanols were also suitable substrates for the present reaction (Table 2, entries 8 and 9). 2-Cyclohexen-1-ol also reacted well to give phenol in excellent yield (Table 2, entry 10). Amide groups on the 4-positions remained intact under the reaction conditions (Table 2, entries 11 and 12). The present dehydrogenation could also be scaled up; for the 10 mmol-scale dehydrogenation of **1a**, the amount of the catalyst could be reduced to 1 mol%, and **2a** was obtained in 90% isolated yield (Table 2, entry 5).

**Table 2:** Substrate scope for the dehydrogenation of cyclohexanols with Au_9_Pd_1_/LDH^*a*^

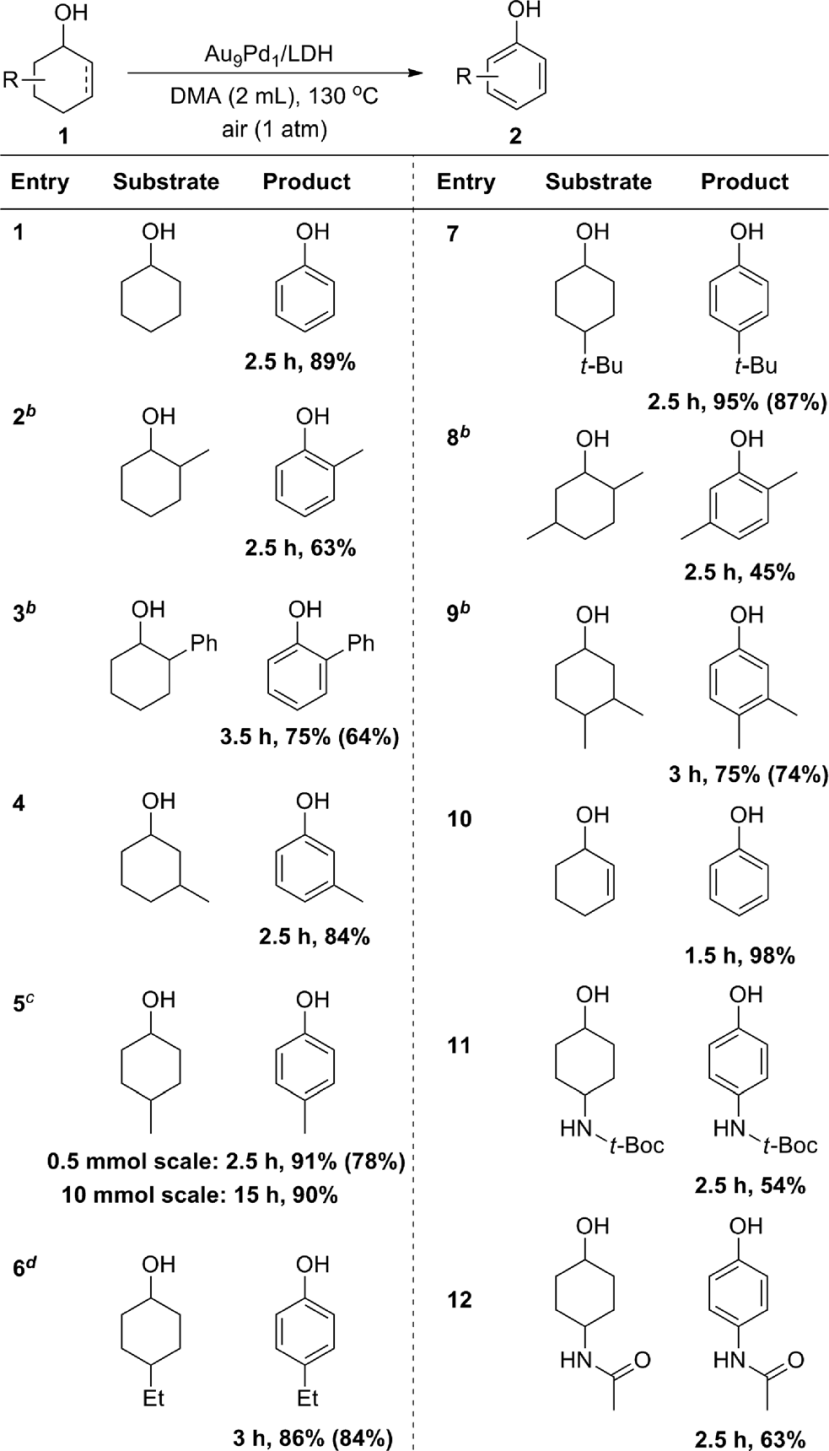

^*a*^Reaction conditions: **1** (0.5 mmol), Au_9_Pd_1_/LDH (total amount of metals: 3.6 mol%), DMA (2 mL), 130 °C, air (1 atm). Conversion and yields were determined by GC analysis. The values in the parentheses are the isolated yields.

Cyclohexanones could also be utilized as starting materials for the synthesis of phenols, and simple cyclohexanone and its derivatives with various substituents at 2-, 3- and 4-positions were all suitable substrates (Table 3). Disubstituted phenols could also be synthesized starting from the corresponding disubstituted cyclohexanones (Table 3, entry 8). In the case of a glycol-protected cyclohexanone, the dehydrogenation proceeded without deprotection of the carbonyl group (Table 3, entry 9). In addition, cyclohexanones substituted with an ester, amide, or alkoxy group were dehydrogenated efficiently without hydrolytic decomposition of these functional groups (Table 3, entries 10–12). Enones, such as 2-cyclohexen-1-one and carvone, could also be utilized as substrates for the present dehydrogenation (Table 3, entries 13 and 14).

**Table 3:** Substrate scope for the dehydrogenation of cyclohexanones with Au_9_Pd_1_/LDH^*a*^

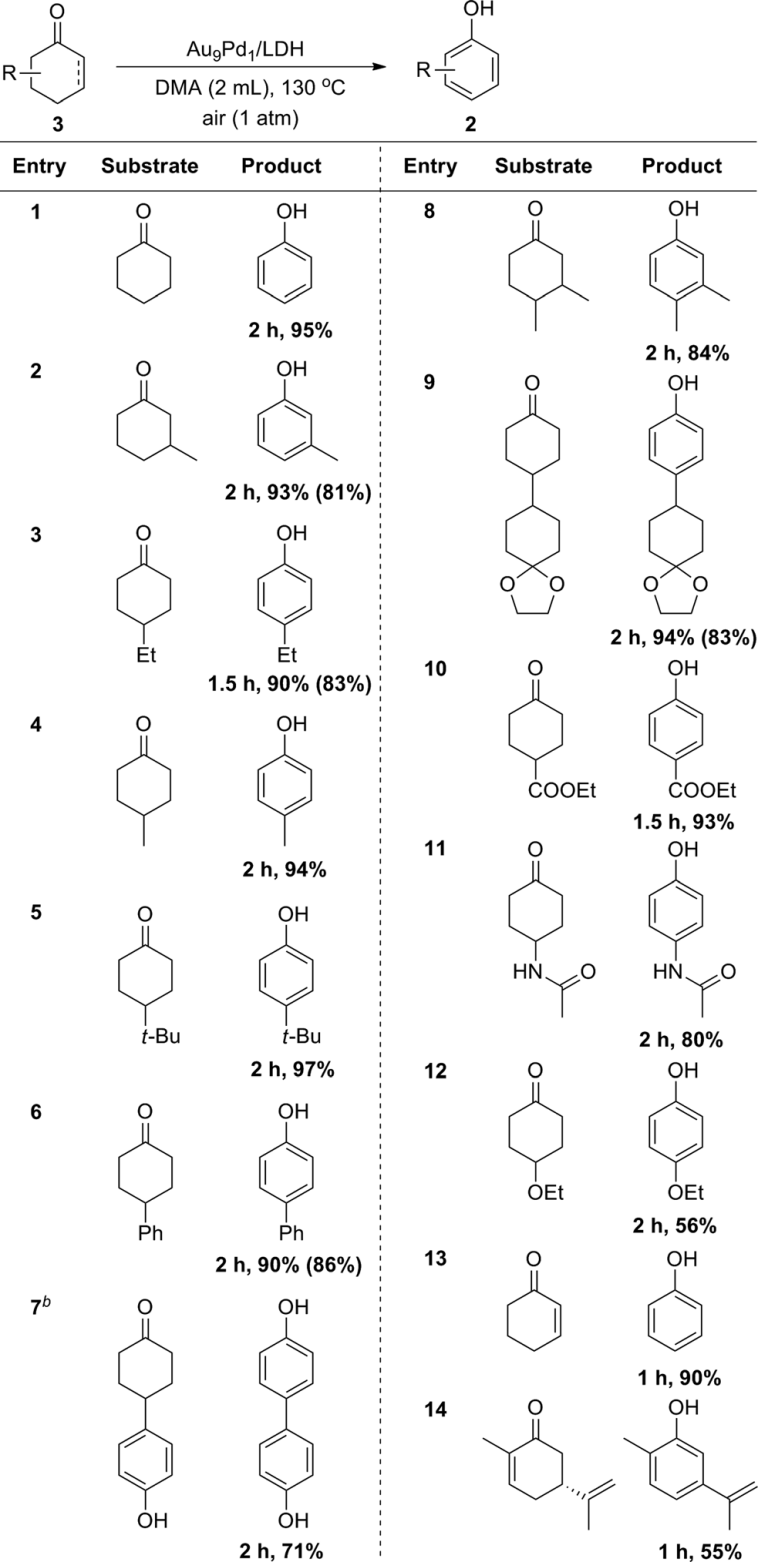

^*a*^Reaction conditions: **3** (0.5 mmol), Au_9_Pd_1_/LDH (total amount of metals: 3.6 mol%), DMA (2 mL), 130 °C, air (1 atm). Conversion and yields were determined by GC analysis. The values in the parentheses are the isolated yields.

Recently, Pd-catalyzed dehydrogenative aromatization has emerged as an attractive method for the synthesis of *N*-substituted anilines from cyclohexanones and amines.^19^ Encouraged by the unique activity of Au_9_Pd_1_/LDH for the dehydrogenative aromatization of cyclohexanols and cyclohexanones, we also synthesized various *N*-substituted anilines using the dehydrogenative aromatization strategy. Under reaction conditions similar to those used for the aforementioned dehydrogenation, cyclohexanones with various substituents on the cyclohexyl rings were efficiently reacted with various alkyl and benzylamines to give the corresponding *N*-substituted anilines (Table 4).

**Table 4:** Substrate scope of the *N*-arylation of amines with cyclohexanones^*a*^

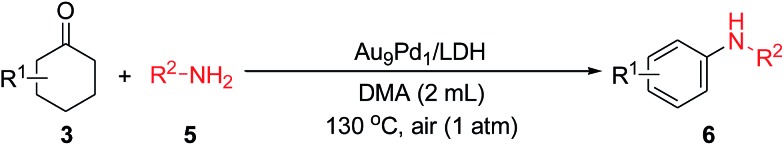
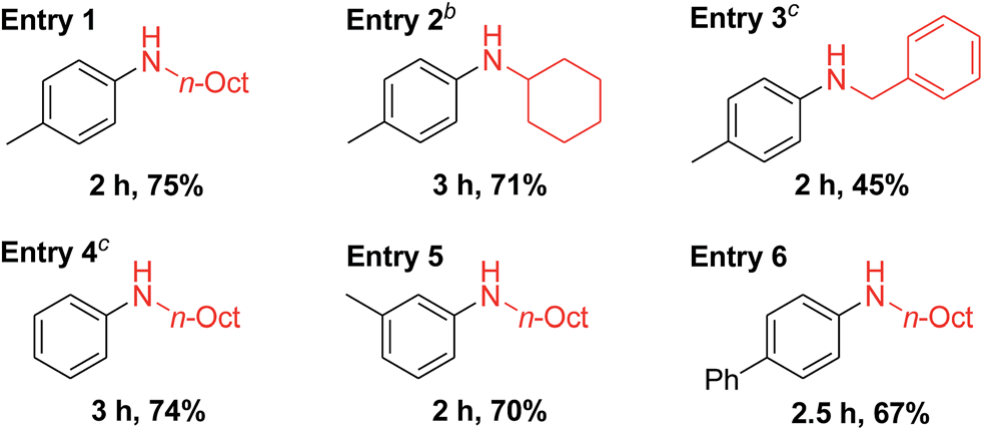

^*a*^Reaction conditions: **3** (1.0 mmol), **5** (0.5 mmol), Au_9_Pd_1_/LDH (total amount of metals: 3.6 mol%), DMA (2 mL), 130 °C, air (1 atm). Conversion and yields were determined by GC analysis.

^*b*^Au_4_Pd_1_/LDH (total amount of metals: 3.6 mol%).

^*c*^ **3** (2.5 mmol).

**Scheme 2:**
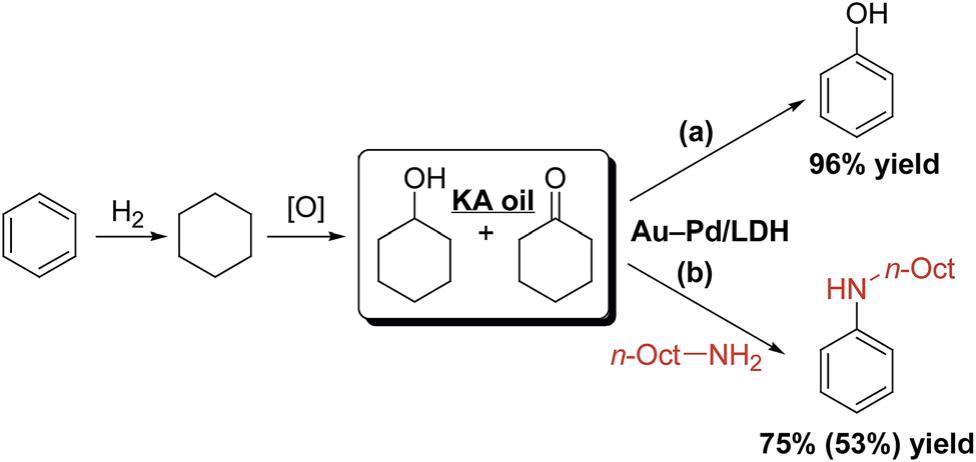
Syntheses of phenol and *N*-octylaniline starting from KA oil. Reaction conditions: (a) **1b** (0.5 mmol), **3b** (0.5 mmol), DMA (2 mL), 130 °C, air (1 atm), 4 h. (b) **1b** (0.5 mmol), **3b** (0.5 mmol), *n*-octylamine (0.5 mmol), DMA (1 mL), mesitylene (1 mL), 130 °C, air (1 atm), 3 h. Yields were determined by GC analysis. The parenthetical value is the isolated yield.

To further demonstrate the potential practical applicability of the present Au_9_Pd_1_/LDH-catalyzed dehydrogenative aromatization, we attempted to synthesize phenol and anilines directly from KA oil. When a 1 : 1 mixture of cyclohexanol and cyclohexanone was used as the starting material, 96% yield of phenol was obtained (Scheme 2a). In addition, KA oil was reacted with *n*-octylamine to give *N*-octylaniline in high yield (Scheme 2b).

### Mechanistic studies

**Fig. 4:**
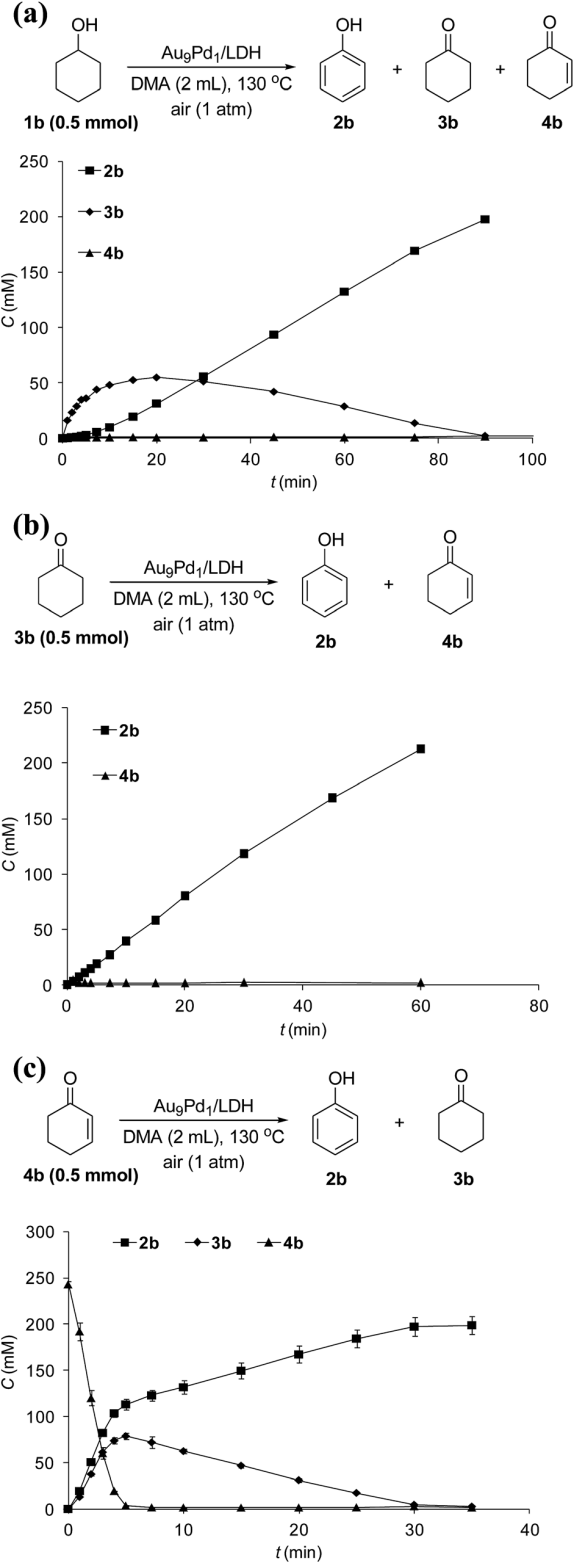
Reaction profiles of dehydrogenations starting from (a) **1b**, (b) **3b**, and (c) **4b**. Reaction conditions: substrates (0.5 mmol), Au_9_Pd_1_/LDH (total amount of metals: 3.6 mol%), DMA (2 mL), 130 °C, air (1 atm). GC yields are shown here.

The reaction profile for the Au_9_Pd_1_/LDH-catalyzed oxidative dehydrogenation of cyclohexanol (**1b**) showed that cyclohexanone (**3b**) was initially produced, followed by the dehydrogenation of **3b** to 2-cyclohexen-1-one (**4b**); the further dehydrogenation of **4b** gave phenol (**2b**) as the final product (Fig. 4a). The dehydrogenation starting from **3b** also gave **2b** in high yield, which suggests the reaction proceeded through **3b** as the intermediate (Fig. 4b). The reaction profile for the dehydrogenation starting from **4b** revealed that **2b** was produced through the disproportionation of **4b** (Fig. 4c). Furthermore, the time course of the dehydrogenation starting from **3b** showed that only a trace amount of **4b** was detected throughout the reaction, which indicates that the disproportionation of **4b** is much faster than the dehydrogenation of **3b** to **4b** (Fig. 4b).

**Fig. 5:**
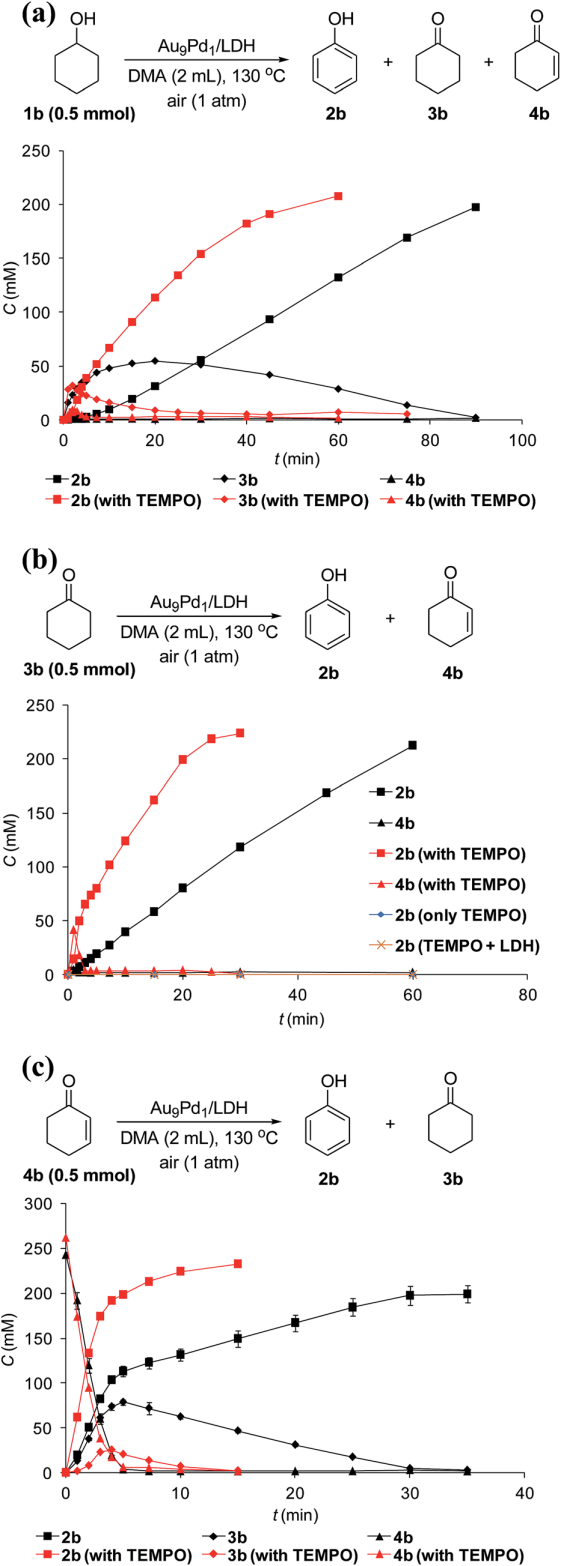
Effects of TEMPO on dehydrogenations starting from (a) **1b**, (b) **3b**, and (c) **4b**. Reaction conditions: substrates (0.5 mmol), Au_9_Pd_1_/LDH (total amount of metals: 3.6 mol%), DMA (2 mL), 130 °C, air (1 atm). GC yields are shown here.

To verify whether the dehydrogenation proceeded through radical intermediates, the Au_9_Pd_1_/LDH-catalyzed dehydrogenation of **1b** was carried out in the presence of a stoichiometric amount of a radical scavenger, 2,2,6,6-tetramethylpiperidine 1-oxyl (TEMPO). In this case, the reaction was not suppressed, but was substantially promoted by the addition of TEMPO and the formation of 1-hydroxy-2,2,6,6-tetramethylpiperidine (TEMPOH) was observed (Fig. 5a). Similarly, the dehydrogenations starting from **3b** and **4b** were both accelerated in the presence of TEMPO, with the concomitant formation of TEMPOH (Fig. 5b and c). Furthermore, control experiments showed that TEMPO only or LDH together with TEMPO (in the absence of catalysts) could not promote the dehydrogenation of **3b** (Fig. 5b). All these results indicate that the involvement of radical intermediates is unlikely in the present transformation. TEMPO has been reported to act as a one-electron oxidant to abstract a hydrogen atom from metal hydride species, where TEMPO itself is reduced to TEMPOH.^20^,^21^ Therefore, the aforementioned experimental results strongly support the involvement of metal hydride species such as Au–H^20^ and Pd–H^21^ in the consecutive dehydrogenations; the reactions were promoted by the oxidation of these metal hydride species by TEMPO. In particular, for the initial stage of the Au_9_Pd_1_/LDH-catalyzed disproportionation of **4b**, the formation of **3b** was substantially suppressed by the addition of TEMPO compared to the reaction in the absence of TEMPO (Fig. 5c, Table S5,† entries 3 and 4). These results suggest that the ability for the oxidation of the metal hydride species (Pd–H) increases in the order O_2_ < **4b** < TEMPO.

As shown in Table 1, for the oxidative dehydrogenation of **1a**, either Au/LDH or Pd/LDH could promote the dehydrogenation of **1a** to **3a** (Table 1, entries 1 and 6); however, their substrate conversions were much lower than that achieved when Au_9_Pd_1_/LDH was used as the catalyst (Table 1, entry 2). These results indicate that both Pd and Au species in Au_9_Pd_1_/LDH intrinsically possess catalytic activity toward the dehydrogenation of **1a** to **3a** and that the dehydrogenation ability was improved by alloying Au and Pd; this observation is in good agreement with results previously reported by other research groups.^11^

Although Au/LDH did not show catalytic activity for the dehydrogenative aromatization of **3b**, Pd/LDH could promote the transformation into **2b**, albeit in low yield (Fig. S6†). In addition, with respect to the disproportionation of **4b**, Pd/LDH exhibited high catalytic activity, whereas Au/LDH afforded only a small amount of **2b** (Fig. S7†). These results suggest that Pd species in the Au–Pd alloy nanoparticles likely play the main role in the dehydrogenation of **3b** to **4b** and the successive disproportionation of **4b**. In addition, the disproportionation of **4b** using Pd/LDH as the catalyst was promoted by the addition of TEMPO, which suggests the involvement of the Pd–H species in the disproportionation (Table S5,† entries 5 and 6).

**Scheme 3:**
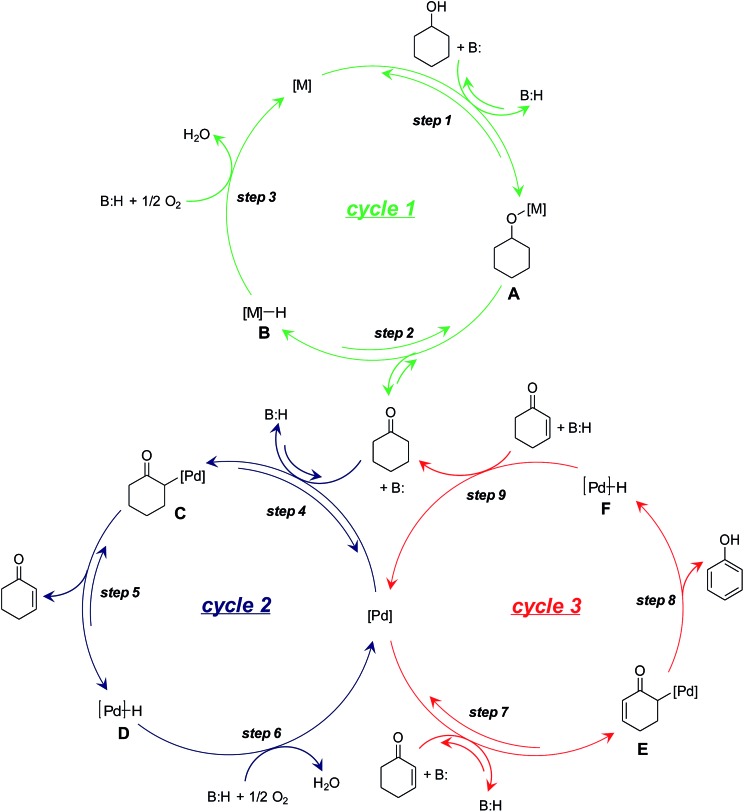
A plausible reaction mechanism. M = Au or Pd.

On the basis of the aforementioned experimental results, we herein propose a plausible reaction mechanism for the dehydrogenation of cyclohexanols to phenols. For the oxidation of cyclohexanols to cyclohexanones (Scheme 3, cycle 1), deprotonative coordination of cyclohexanols to the metal species (either Au or Pd) promoted by LDH generates metal alkoxy species (Scheme 3, cycle 1, step 1), followed by β-H elimination to give cyclohexanones with the concomitant formation of metal hydride species (either Au–H or Pd–H) (Scheme 3, cycle 1, step 2). The metal hydride species can then be oxidized by O_2_ (Scheme 3, cycle 1, step 3). For the dehydrogenation of cyclohexanones to cyclohexenones (Scheme 3, cycle 2), deprotonative coordination of cyclohexanones to Pd with the assistance of basic LDH affords Pd-enolate species (Scheme 3, cycle 2, step 4) followed by β-H elimination to give cyclohexenones (Scheme 3, cycle 2, step 5). The oxidation of Pd–H can then regenerate the catalyst (Scheme 3, cycle 2, step 6).^22^ Finally, the disproportionation of cyclohexenones proceeds through a catalytic cycle similar to that of the dehydrogenation of cyclohexanones to cyclohexenones (Scheme 3, cycle 3). Specifically, LDH-assisted α-C–H cleavage generates Pd-enolate species (Scheme 3, cycle 3, step 7) followed by β-H elimination to give the final phenol products with the concomitant formation of the Pd–H species (Scheme 3, cycle 3, step 8). In the cycle 3, the hydrogen acceptor is cyclohexenones rather than O_2_ (Scheme 3, cycle 3, step 9).

Because of the mechanism of alcohol oxidation by supported Au–Pd alloy nanoparticles has been extensively investigated by other research groups,^11^ we herein mainly focused on the elucidation of the detailed roles of Au–Pd alloy nanoparticles in the dehydrogenation of cyclohexanones to phenols. As previously mentioned, Pd/LDH exhibited significant catalytic activity for the oxidation of **1a** to **3a** (Table 1, entry 6) but did not exhibit catalytic activity for the dehydrogenative aromatization of **3a** to **2a** (Table S2,† entry 6). Furthermore, the disproportionation of **4b** was efficiently promoted by Pd/LDH (Fig. S7†). These results suggest that the Au atoms in Au–Pd alloy nanoparticles play an important assisting role on the dehydrogenation of cyclohexanones to cyclohexenones by Pd (Scheme 3, cycle 2).

In addition, the dehydrogenation of **3b** with Pd/LDH was not accelerated by TEMPO even though TEMPO is known to promote the oxidation of Pd–H species (Table S4,† entries 5 and 6);^21^ this result suggests that the low activity of Pd/LDH for the dehydrogenation of **3b** is not likely due to the inertness of Pd–H species toward oxidation by O_2_ (Scheme 3, cycle 2, step 6) but is instead due to slow β-H elimination (Scheme 3, cycle 2, step 5). The Au species in Au–Pd alloy nanoparticles possibly promote β-H elimination by Pd, likely *via* electronic ligand effects.^9^–^12^ The greater electronegativity of Au compared to that of Pd has been reported to result in a net electron transfer from Pd to Au; thus, the Au species become more electron-rich and the Pd species become more electron-poor.^9^–^12^ Therefore, the β-H elimination step in the dehydrogenation of **3b** to **4b** likely become more favorable with increasing Au/Pd molar ratio of the catalysts. Consequently, the catalytic activity of the Au_*x*_Pd_*y*_/LDH catalysts possibly increased with increasing Au/Pd molar ratio, and thus Au_9_Pd_1_/LDH exhibits much higher catalytic activity than other catalysts toward the present dehydrogenation (Tables 1 and S3†).

**Fig. 6:**
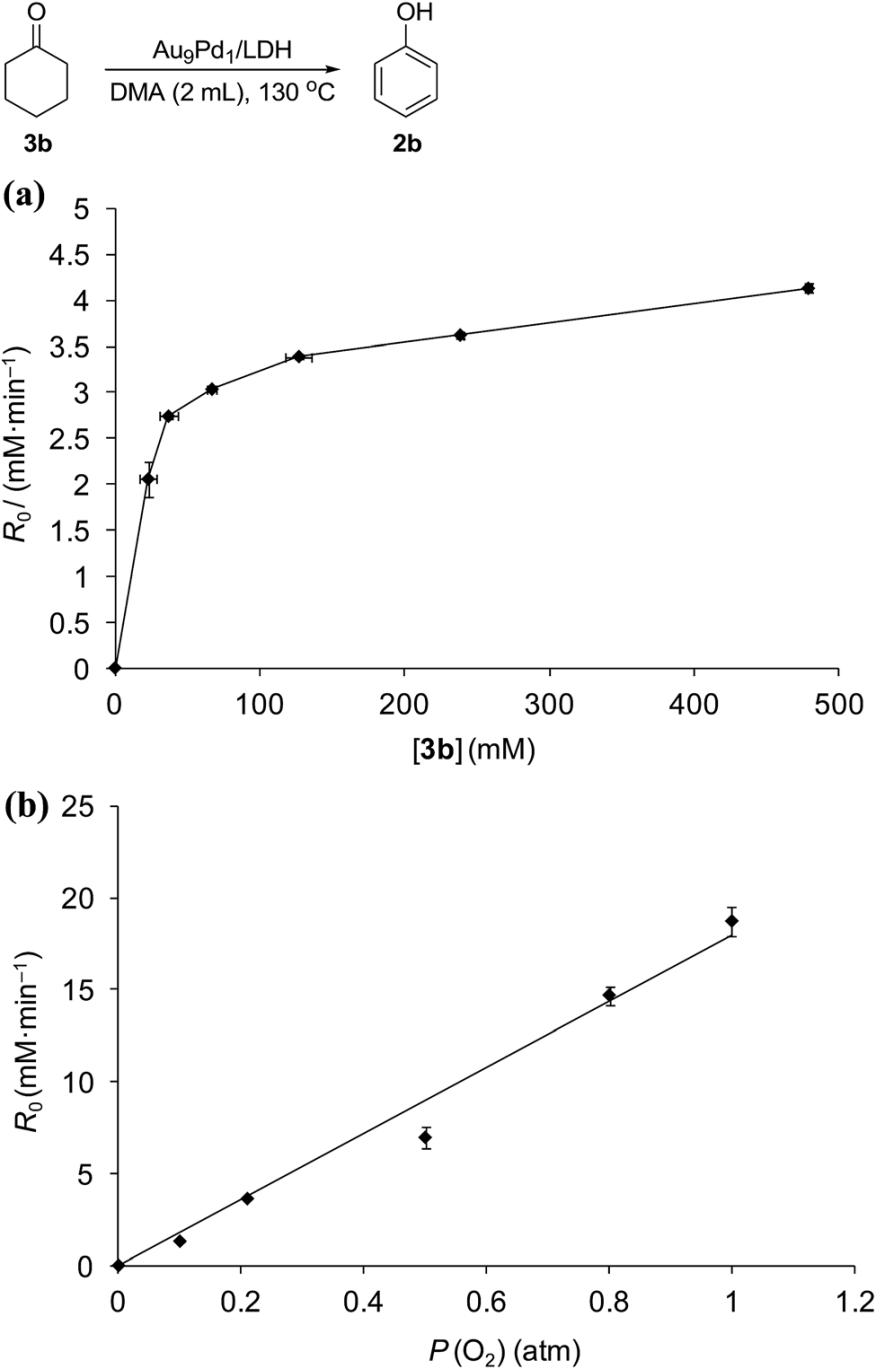
Dependence of reaction rates of the dehydrogenation of **3b** on (a) the concentration of **3b** and (b) the partial pressure of O_2_, line fit: *R*_0_ = 17.9*P* (O_2_), *R*^2^ = 0.98. Reaction conditions: (a) **3b** (22.9–478.9 mM), Au_9_Pd_1_/LDH (total amount of metals: 3.6 mol%), DMA (2 mL), 130 °C, air (1 atm); (b) **3b** (250 mM), Au_9_Pd_1_/LDH (total amount of metals: 3.6 mol%), DMA (2 mL), 130 °C, O_2_ (0.1–1 atm). GC yields are shown here.

Kinetic studies on the Au_9_Pd_1_/LDH-catalyzed dehydrogenation of **3b** showed the saturation kinetics of the reaction rate based on the concentration of **3b** (22.9–478.9 mM, Fig. 6a), which indicates that a pre-equilibrium exists between Pd species and Pd-enolate species for cleavage of an α-C–H bond. The dehydrogenation of cyclohexanone-2,2,6,6-*d*_4_ (97% deuterium labeling at the 2- and 6-positions) gave phenol with 65% deuterium labeling at the 2- and 6-positions (Scheme S1†); this deuterium loss also supports the existence of the pre-equilibrium. In addition, the kinetic studies revealed a first-order dependence of the reaction rate on the partial pressure of O_2_ (0.1–1.0 atm, Fig. 6b), which suggests that the oxidation of the Pd–H species is included in the rate-determining step for the dehydrogenation of **3b**. The aforementioned significant promotion effects of TEMPO also suggest that the oxidation of the Pd–H species is included in the rate-determining step.

## Conclusion

We successfully developed a novel heterogeneously-catalyzed oxidative dehydrogenation reaction for the conversion of cyclohexanols and cyclohexanones into phenols using Au_9_Pd_1_/LDH. Monometallic Au or Pd supported on LDH exhibited almost no activity toward the dehydrogenation, whereas marked enhancement of the catalytic activity was observed for alloyed Au and Pd. Mechanistic studies showed that β-H elimination from Pd-enolate species is accelerated by Au species, likely *via* electronic ligand effects; this acceleration is the key to achieve the high activity of Au_9_Pd_1_/LDH. The basicity of LDH also plays a deterministic role in the present dehydrogenation through its assistance in the deprotonation steps. Au_9_Pd_1_/LDH functioned as a truly heterogeneous catalyst and could be reused at least 10 times with retention of its high catalytic performance. The present dehydrogenation utilizes O_2_ in air as the terminal oxidant and generates water as the only by-product, which highlights the environmentally benign nature of the present transformation. The substrate scope was very broad with respect to both cyclohexanols and cyclohexanones, and *N*-substituted anilines could also be synthesized starting from cyclohexanones and amines. Furthermore, the present catalytic system could be scaled up and applied to the direct conversion of KA oil to phenol. Thus, we demonstrated the potential practical applicability of the present catalyst system for the synthesis of phenol from benzene in industrial processes.

## Experimental section

### Instruments and reagents

Gas chromatography (GC) analyses were performed on a Shimadzu GC-2014 equipped with a flame ionization detector (FID) and an InertCap-5 or a TC-WAX capillary column. GC mass spectrometry (GC-MS) spectra were recorded on a Shimadzu GCMS-QP2010 equipped with an InertCap 5 capillary column at an ionization voltage of 70 eV. Liquid-state NMR spectra were recorded on a JEOL JNM-ECA-500 spectrometer. ^1^H and ^13^C NMR spectra were measured at 495.1 and 124.5 MHz, respectively, using tetramethylsilane (TMS) as an internal reference (*δ* = 0 ppm). ICP-AES analyses were performed on a Shimadzu ICPS-8100. TEM observations were performed on JEOL JEM-2010HC. HAADF-STEM and EDS images were obtained using JEOL JEM-ARM 200F operating at 200 kV. XRD patterns were collected on a Rigaku SmartLab diffractometer (Cu_Kα_, *λ* = 1.5405 Å, 45 kV, 200 mA). UV-Vis diffuse reflectance spectra were recorded on a Jasco V-570DS. Mg–Al LDH (Mg_6_Al_2_(OH)_16_CO_3_·4H_2_O, BET surface area: 51 m^2^ g^–1^, Tomita Pharmaceutical Co., Ltd.), CeO_2_ (BET surface area: 111 m^2^ g^–1^, Cat. no. 544841-25G, Aldrich), Al_2_O_3_ (BET surface area: 160 m^2^ g^–1^, Cat. no. KHS-24, Sumitomo Chemical), MgO (BET surface area: 28 m^2^ g^–1^, Cat. No. P0082, Ube Industries Ltd.), and TiO_2_ (BET surface area: 316 m^2^ g^–1^, Cat. No. ST-01, Ishihara Sangyo Kaisya) were acquired from commercial sources. Solvents and substrates were obtained from Kanto Chemical, TCI, Wako, or Aldrich (reagent grade) and were purified before use (if required).^23^

### Preparation of catalysts

Au–Pd/LDH with different Au/Pd ratios, such as Au_9_Pd_1_/LDH, was typically prepared as follows. First, Mg–Al LDH (2.0 g) was added to 60 mL of an aqueous solution of HAuCl_4_·4H_2_O (7.50 mM), PdCl_2_ (0.83 mM), and KCl (2 equiv. with respect to PdCl_2_, 1.67 mM). The resulting slurry was then stirred vigorously at room temperature for 12 h. The solid was then filtered off, washed with water (3 L), and dried *in vacuo* to afford the supported hydroxide precursor. The hydroxide precursor was redispersed in 50 mL water and reduced with NaBH_4_ (70 mg). The resulting slurry was then stirred vigorously at room temperature for 2 h. The solid was then filtered off, washed with water (2 L), and dried *in vacuo* overnight, giving Au_9_Pd_1_/LDH as a dark-gray powder (Au content: 0.148 mmol g^–1^, Pd content: 0.024 mmol g^–1^). Au_4_Pd_1_/LDH (Au content: 0.134 mmol g^–1^, Pd content: 0.051 mmol g^–1^), Au_1_Pd_1_/LDH (Au content: 0.091 mmol g^–1^, Pd content: 0.122 mmol g^–1^), and Au_1_Pd_4_/LDH (Au content: 0.044 mmol g^–1^, Pd content: 0.192 mmol g^–1^) were prepared *via* the same method used to prepare Au_9_Pd_1_/LDH. Preparation methods for other catalysts are described in the ESI.†

### Catalytic dehydrogenation

In a typical dehydrogenation procedure, Au–Pd/LDH (105 mg, total amount of metals: 3.6 mol%), cyclohexanone (0.5 mmol), DMA (2.0 mL), and a Teflon-coated magnetic stir bar were successively placed in a Pyrex glass reactor (volume: *ca.* 20 mL) and the reaction mixture was vigorously stirred at 50 °C under 1 atm of air. After the reaction was completed, an internal standard (diphenyl) was added to the reaction mixture and the conversion of cyclohexanone and the yield of phenol were determined by GC analysis. In cases where phenol products were isolated, an internal standard was not added. After the reaction, the catalyst was filtered off (>95% recovery). EtOAc (15 mL) and *n*-hexane (5 mL) were then added to the filtrate, which was washed with brine (10 mL) three times. The organic phase was collected and evaporated to remove solvents. The crude product was subjected to column chromatography on silica gel (for phenols, *n*-hexane/acetone was typically used as an eluent; for *N*-octylaniline, *n*-hexane/ethylacetate was used), giving the pure phenols. The products were identified by GC-MS and NMR (^1^H and ^13^C) analyses. The recovered catalyst was washed with water and ethanol (or 0.1 M NaOH in 1 : 1 water/ethanol) before being used in the reuse experiment.

## Supplementary Material

Supplementary information
