# Supplementary material for: Au–Pd alloy nanoparticles supported on layered double hydroxide for heterogeneously catalyzed aerobic oxidative dehydrogenation of cyclohexanols and cyclohexanones to phenols
Source: Chem Sci. 2016 May 6;7(8):5371–83. doi: 10.1039/c6sc00874g (PMC6020756; doi:10.1039/c6sc00874g)
Supplement: Supplementary file 1 [file SC-007-C6SC00874G-s001.pdf]

Electronic Supplementary Material (ESI) for Chemical Science.
This journal is © The Royal Society of Chemistry 2016

Electronic Supplementary Information (ESI) for

Au-Pd alloy nanoparticles supported on layered double hydroxide for heterogeneously catalyzed aerobic oxidative dehydrogenation of cyclohexanols and cyclohexanones to phenols

Xiongjie Jin, Kento Taniguchi, Kazuya Yamaguchi and Noritaka Mizuno*

Department of Applied Chemistry, School of Engineering, The University of Tokyo, 7-3-1 Hongo, Bunkyo-ku, Tokyo 113-8656
E-mail: tmizuno@mail.ecc.u-tokyo.ac.jp

Experimental

Preparation of catalysts

Au/LDH was prepared as follows. First, Mg–Al LDH (2.0 g) was added to 60 mL aqueous solution of $HAuCl_4{\cdot}4H_2O$ (8.3 mM). Then the resulting slurry was stirred vigorously at room temperature for 5 min, followed by the addition of 30% ammonia solution (0.15 mL). The slurry was further stirred vigorously at room temperature for 14 h. The solid was then filtered off, washed with water (3 L), and dried in vacuo to afford the supported hydroxide precursor. The hydroxide precursor was redispersed in 50 mL water, and reduced with $NaBH_4$ (70 mg). Then the resulting slurry was stirred vigorously at room temperature for 2 h. The solid was then filtered off, washed with water (2 L), and dried in vacuo overnight, giving Au/LDH as a purple powder (Au content: 0.205 mmol g^{-1}).

Pd/LDH was prepared as follows. First, Mg–Al LDH (2.0 g) was added to 60 mL aqueous solution of $PdCl_2$ (8.3 mM) and KCl (2 equiv with respect to $PdCl_2$, 16.6 mM). Then the resulting slurry was stirred vigorously at room temperature for 5 min. The pH of the solution was quickly adjusted to 10 by addition of an aqueous solution of NaOH (1.0 M), and the resulting slurry was further stirred for 20 h. The solid was then filtered off, washed with water (3 L), and dried in vacuo to afford the supported hydroxide precursor. The hydroxide precursor was redispersed in 50 mL water, and reduced with $NaBH_4$ (70 mg). Then the resulting slurry was stirred vigorously at room temperature for 2 h. The solid was then filtered off, washed with water (2 L), and dried in vacuo

overnight, giving Pd/LDH as a dark grey powder (Pd content: 0.237 mmol g^{-1}).

Au_9Pd_1/Al_2O_3, Au_9Pd_1/TiO_2, Au_9Pd_1/CeO_2, and Au_4Pd_1/Al_2O_3 were prepared according to the literature procedures.[17i] Au_9Pd_1/MgO was prepared via the same method used to prepare Au_9Pd_1/LDH. The average metal nanoparticle sizes and Au and Pd contents are show in Table S1.

Synthesis of cyclohexanone-2,2,6,6-d_4

Cyclohexanone-2,2,6,6-d_4 was prepared according to the literature procedures.[S1] Into a pyrex grass reactor (volume: ca. 50 mL), cyclohexanone (9.7 mmol, 0.95 g), Na_2CO_3 (4.8 mmol, 0.51 g), D_2O (12 mL), and a Teflon-coated magnetic stir bar were successively placed, and the mixture was vigorouslly stirred at 120 °C for 18 h. After the reaction, the crude mixture was extracted with Et_2O (20 mL) 3 times. The organic phase was gatherd, and dried with N_2SO_4. Then, evaporation of the solvent gave cyclohexanone-2,2,6,6-d_4 as light yellow oil (97% deuterium labeling at the 2- and 6-positions). ^{1}H NMR (495.1 MHz, $CDCl_3$, TMS): δ 1.70−1.75 (m, 2H), 1.84−1.86 (m, 4H).

Additional reference

(S1) Gigant, N.; Bäckvall, J.-E. *Chem. Eur. J.* **2014**, *20*, 5890.

Spectral data of phenols and *N*-substituted anilines

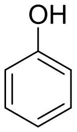

(CAS number: 108-95-2)

phenol: MS (EI): *m*/*z* (%) : 94 (100) [*M*+], 66 (17), 65 (13), 55 (4).

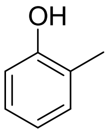

(CAS number: 95-48-7)

2-methylphenol: MS (EI): *m*/*z* (%) : 108 (100) [*M*+], 107 (80), 90 (19), 89 (11), 80 (12), 79 (26), 77 (23).

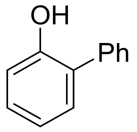

(CAS number: 90-43-7)

2-phenylphenol: ^{1}H NMR (495.1 MHz, $CDCl_3$, TMS): *δ* 5.26 (s, 1H), 6.97−7.01 (m, 2H), 7.23−7.28 (m, 2H), 7.37−7.41 (m, 1H), 7.45−7.50 (m, 4H). $^{13}C\{^{1}H\}$ NMR (124.5 MHz, $CDCl_3$, TMS): *δ* 115.94, 120.96, 128.00, 128.24, 129.22, 129.28, 129.39, 130.37, 137.19, 152.53. MS (EI): *m*/*z* (%) : 170 (100) [M^+], 169 (66), 142 (13), 141 (36), 139 (12), 115 (30), 89 (8), 77 (4), 63 (6), 51 (4).

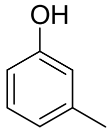

(CAS number: 108-39-4)

3-methylphenol: ^{1}H NMR (495.1 MHz, $CDCl_3$, TMS): *δ* 2.31 (s, 3H), 4.87 (s, 1H), 6.62−6.66 (m, 2H), 6.74−6.76 (m, 1H), 7.11−7.14 (m, 1H). $^{13}C\{^{1}H\}$ NMR (124.5 MHz, $CDCl_3$, TMS): *δ* 21.49, 112.40, 116.13, 121.73, 129.56, 139.97, 155.57. MS (EI): *m*/*z* (%) : 108 (100) [M^+], 107 (87), 90 (10), 80 (10), 79 (25), 77 (21).

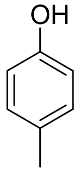

(CAS number: 106-44-5)

4-methylphenol: ^{1}H NMR (495.1 MHz, $CDCl_3$, TMS): *δ* 2.27 (s, 3H), 4.92 (brs, 1H), 6.72−6.74 (m, 2H), 7.02−7.03 (m, 2H). $^{13}C\{^{1}H\}$ NMR (124.5 MHz, $CDCl_3$, TMS): *δ* 20.60, 115.22, 130.08, 130.19, 153.38. MS (EI): *m*/*z* (%) : 108 (90) [M^+], 107 (100), 80 (10), 79 (21), 77 (27), 53 (11), 51 (12).

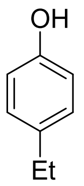

(CAS number: 123-07-9)

4-ethylphenol: ^{1}H NMR (495.1 MHz, $CDCl_3$, TMS): δ 1.20 (t, J = 7.7 Hz, 3H), 2.57 (q, J = 7.6 Hz, 2H), 5.00 (brs, 1H), 6.74−6.77 (m, 2H), 7.05−7.07 (m, 2H). ^{13}C{^{1}H} NMR (124.5 MHz, $CDCl_3$, TMS): δ 16.01, 28.10, 115.26, 129.03, 136.68, 153.49. MS (EI): m/z (%) : 122 (40) [M^+], 107 (100), 77 (14).

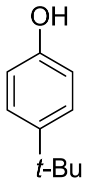

(CAS number: 98-54-4)

4-*t*-buthylphenol: ^{1}H NMR (495.1 MHz, $CDCl_3$, TMS): δ 1.28−1.29 (m, 9H), 4.89 (brs, 1H), 6.75−6.78 (m, 2H), 7.24−7.28 (m, 2H). ^{13}C{^{1}H} NMR (124.5 MHz, $CDCl_3$, TMS): δ 31.67, 34.21, 114.90, 126.58, 143.68, 153.24. MS (EI): m/z (%) : 150 (22) [M^+], 136 (10), 135 (100), 107 (38), 95 (13), 91 (9), 77 (9), 65 (6), 51 (3).

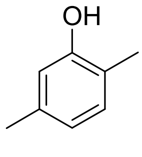

(CAS number: 95-87-4)

2,5-dimethylphenol: MS (EI): m/z (%) : 122 (100) [M^+], 121 (36), 107 (90), 91 (16), 79 (15), 77 (22).

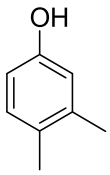

(CAS number: 95-65-8)

3,4-dimethylphenol: ^{1}H NMR (495.1 MHz, $CDCl_3$, TMS): δ 2.18 (s, 3H), 2.21 (s, 3H), 4.75 (brs, 1H), 6.57−6.58 (m, 1H), 6.64 (s, 1H), 6.98 (d, J = 8.4 Hz, 1H). $^{13}C\{^{1}H\}$ NMR (124.5 MHz, $CDCl_3$, TMS): δ 18.91, 20.01, 112.44, 116.70, 128.76, 130.61, 138.11, 153.57. MS (EI): *m*/*z* (%) : 122 (86) [M^{+}], 121 (42), 107 (100), 91 (12), 77 (18).

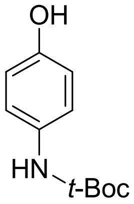

(CAS number: 54840-15-2)

4-(*N*-*t*-butoxycarbonylamino)phenol: MS (EI): *m*/*z* (%) : 209 (20) [M^{+}], 153 (98), 135 (11), 109 (100), 108 (11), 80 (13), 59 (17), 57 (59).

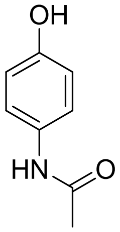

(CAS number: 103-90-2)

4-acetamidophenol: MS (EI): *m*/*z* (%) : 151 (31) [M^{+}], 109 (100), 108 (14), 81 (19), 80 (28), 53 (17), 52 (12).

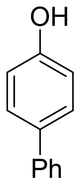

(CAS number: 92-69-3)

4-phenylphenol: ^{1}H NMR (495.1 MHz, $CDCl_3$, TMS): δ 4.96 (brs, 1H), 6.89−6.92 (m, 2H), 7.29−7.32 (m, 1H), 7.39−7.43 (m, 2H), 7.47−7.49 (m, 2H), 7.53−7.55 (m, 2H). $^{13}C\{^{1}H\}$ NMR

(124.5 MHz, $CDCl_3$, TMS): δ 115.78, 126.86, 128.54, 128.87, 134.16, 140.89, 155.20. MS (EI): *m*/*z* (%) : 171 (13), 170 (100) [M^+], 141 (25), 115 (18).

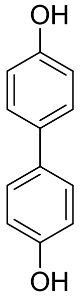

(CAS number: 92-88-6)

4,4'-dihydroxybiphenyl: MS (EI): *m*/*z* (%) : 187 (13), 186 (100) [M^+], 157 (12), 128 (6), 93 (8).

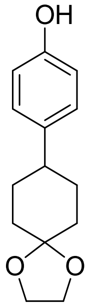

(CAS number: 142256-87-9)

4-(1,4-dioxaspiro[4.5]dec-8-yl)phenol: ^{1}H NMR (495.1 MHz, $CDCl_3$, TMS): δ 1.66−1.87 (m, 8H), 2.47−2.53 (m, 1H), 3.99 (s, 4H), 5.38 (s, 1H), 6.74−6.77 (m, 2H), 7.08−7.10 (m, 2H). $^{13}C\{^1H\}$ NMR (124.5 MHz, $CDCl_3$, TMS): δ 31.84, 35.21, 42.49, 64.35, 64.42, 108.86, 115.25, 128.01, 138.78, 154.01. MS (EI): *m*/*z* (%) : 234 (11) [M^+], 133 (2), 120 (18), 107 (3), 100 (6), 99 (100), 91 (2), 87 (9), 86 (7), 77 (1), 55 (5).

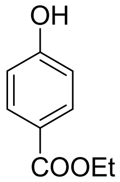

(CAS number: 120-47-8)

4-(ethoxycarbonyl)phenol: MS (EI): *m*/*z* (%) : 166 (24) [M^+], 138 (26), 122 (11), 121 (100), 93 (14), 65 (8).

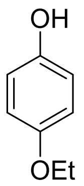

(CAS number: 622-62-8)

4-ethoxyphenol: MS (EI): *m*/*z* (%) : 138 (59) [M^{+}], 110 (100), 109 (13), 82 (10), 81 (12).

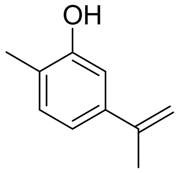

(CAS number: 56423-47-3)

2-methyl-5-(1-methylethenyl)phenol: MS (EI): *m*/*z* (%) : 149 (11), 148 (100) [M^{+}], 147 (15), 133 (43), 108 (29), 107 (14), 105 (22), 91 (10), 77 (13).

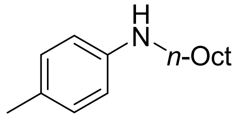

(CAS number: 18977-67-8)

4-methyl-*N*-octylaniline: MS (EI): *m*/*z* (%) : 219 (13) [M^{+}], 121 (9), 120 (100), 91 (9), 77 (4), 65 (4).

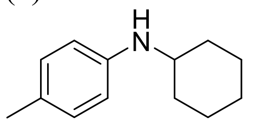

(CAS number: 10386-93-3)

***N*-cyclohexyl-4-methylaniline:** MS (EI): *m*/*z* (%) : 189 (39) [M^{+}], 147 (14), 146 (100), 133 (16), 132 (14), 131 (18), 120 (9), 118 (9), 107 (11), 106 (18), 91 (16), 77 (10), 65 (9), 55 (13).

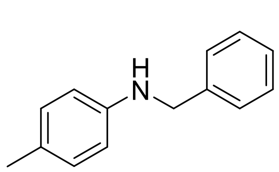

(CAS number: 5405-15-2)

4-methyl- *N*-benzylaniline: MS (EI): *m*/*z* (%) : 197 (35) [M^{+}], 196 (12), 120 (13), 91 (100), 77 (19), 65 (34), 51 (10).

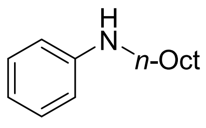

(CAS number: 3007-71-4)

***N*-octylaniline:** ^{1}H NMR (495.1 MHz, $CDCl_3$, TMS): δ 0.87−0.89 (m, 3H), 1.27−1.30 (m, 8H), 1.37−1.40 (m, 2H), 1.58−1.63 (m, 2H), 3.07−3.11 (m, 2H), 3.58 (brs, 1H), 6.59−6.60 (m, 2H), 6.59−6.69 (m, 1H), 7.15−7.18 (m, 2H). $^{13}C\{^{1}H\}$ NMR (124.5 MHz, $CDCl_3$, TMS): δ 14.26, 22.80, 27.33, 29.41, 29.56, 29.72, 31.97, 44.13, 112.81, 117.18, 129.34, 148.67. MS (EI): *m*/*z* (%) : 205 (8) [M^{+}], 106 (100), 93 (4), 77 (13), 65 (4), 55 (4), 51 (4).

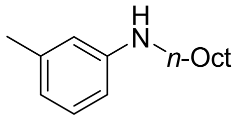

(CAS number: 57154-23-1)

3-methyl-*N*-octylaniline: MS (EI): *m*/*z* (%) : 219 (10) [M^{+}], 120 (100), 91 (15), 77 (6), 65 (8), 55 (3).

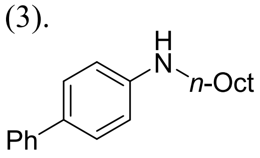

(CAS number: 1013913-33-1)

***N*-octyl-(1,1'-biphenyl)-4-amine:** MS (EI): *m*/*z* (%) : 281 (35) [M^{+}], 183 (16), 182 (100), 169 (4), 152 (13), 115 (4), 77 (3), 55 (5)

Table S1 Metal contents and average particle sizes of various supported metal nanoparticle catalysts

catalyst	Au content ($mmol\cdot g^{-1}$)	Pd content ($mmol\cdot g^{-1}$)	average size (nm)	standard deviation (nm)
Au_9Pd_1/LDH	0.148	0.024	3.2	0.8
Au_4Pd_1/LDH	0.134	0.051	2.6	0.6
Au_1Pd_1/LDH	0.091	0.122	2.8	0.7
Au_1Pd_4/LDH	0.044	0.192	2.8	0.7
Au/LDH	0.205	–	5.3	1.4
Pd/LDH	–	0.237	2.9	0.7
Au_9Pd_1/Al_2O_3	0.180	0.023	4.6	1.6
Au_4Pd_1/Al_2O_3	0.137	0.042	1.7	0.4
Au_9Pd_1/TiO_2	0.159	0.026	3.4	0.8
Au_9Pd_1/MgO	0.076	0.020	3.4	1.1
Au_9Pd_1/CeO_2	0.205	0.022	3.9	1.2

(N = 200. For Au_9Pd_1/LDH, N = 400)

Table S2 Solvent effect on oxidative dehydrogenation of 4-methylcyclohexanol (**1a**) to 4-methylphenol (**2a**)[a]

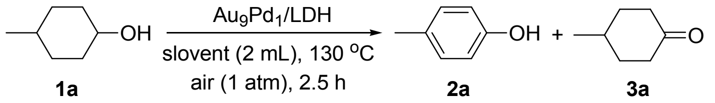

entry	solvent	conv. (%)	yield (%) 2a	yield (%) 3a
**1**	**DMA**	**94**	**91**	**2**
2	DMF	51	29	15
3	NMP	85	22	23
4	DMSO	<1	nd	nd
5	monochlorobenzene	68	nd	12
6	mesitylene	>99	33	27

[a]Reaction conditions: **1a** (0.5 mmol), Au_9Pd_1/LDH (total amount of metals: 3.6 mol%), solvent (2 mL), 130 °C, air (1 atm), 2.5 h. Conversion and yields were determined by GC analysis. DMF = *N*,*N*-dimethylformamide. NMP = *N*-methylpyrrolidone. DMSO = dimethylsulfoxide.

Table S3 Dehydrogenative aromatization of 4-methylcyclohexanone (**3a**) to 4-methylphenol (**2a**) with various catalysts[a]

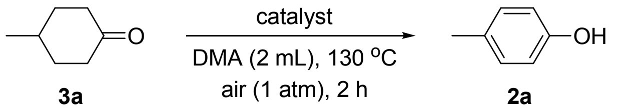

entry	catalyst	conv. (%)	yield (%)	Au/Pd[b]
1	Au/LDH	10	2	3.6/0
**2**	**Au_9Pd_1/LDH**	**>99**	**94**	**3.1/0.5**
3	Au_4Pd_1/LDH	>99	88	2.6/1.0
4	Au_1Pd_1/LDH	36	13	1.5/2.1
5	Au_1Pd_4/LDH	23	nd	0.7/2.9
6	Pd/LDH	20	nd	0/3.6
7	Au_9Pd_1/Al_2O_3	7	nd	3.2/0.4
8	Au_9Pd_1/TiO_2	37	nd	3.1/0.5
9	Au_9Pd_1/CeO_2	24	2	3.2/0.4
10	Au_9Pd_1/MgO	12	3	2.9/0.7
11	Au_4Pd_1/Al_2O_3	24	4	2.8/0.8
12[c]	$Au_4Pd_1/Al_2O_3 + K_2CO_3$	82	63	2.8/0.8
13[d]	Au_4Pd_1/Al_2O_3 + LDH	37	15	2.8/0.8
14[e]	Au/LDH + Pd/LDH	11	nd	3.1/0.5
15[d]	Au_9Pd_1/TiO_2 + LDH	52	13	3.1/0.5
16[f]	Au_9Pd_1/LDH	<1	nd	3.1/0.5
17	none	23	nd	–

[a]Reaction conditions: **1a** (0.5 mmol), catalyst (total amount of metals: 3.6 mol%), DMA (2 mL), 130 °C, air (1 atm), 2 h. Conversion and yields were determined by GC analysis. [b]Amount of metals (mol%). [c]K_2CO_3 (0.5 mmol). [d]LDH (100 mg). [e]A physical mixture of Au/LDH (3.1 mol%) and Pd/LDH (0.5 mol%). [f]Ar (1 atm).

Table S4 Effects of TEMPO on dehydrogenative aromatization of cyclohexanone (**3b**) to phenol (**2b**)[a]

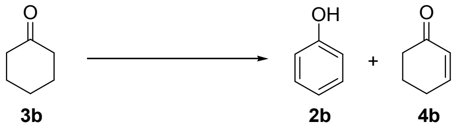

entry	catalyst	additive	conv. (%)	yield (%)	
				**2b**	**4b**
1	Au/LDH	–	20	nd	2
2	Au/LDH	TEMPO	32	1	4
3	Au_9Pd_1/LDH	–	62	49	1
4	Au_9Pd_1/LDH	TEMPO	>99	92	nd
5	Pd/LDH	–	32	12	3
6	Pd/LDH	TEMPO	35	10	4
7	–	TEMPO	10	nd	nd
8[b]	–	TEMPO	4	nd	nd

[a]Reaction conditions: **3b** (0.5 mmol), catalyst (total amount of metals: 3.6 mol%), TEMPO (1 equiv), DMA (2 mL), 130 °C, air (1 atm), 30 min. Conversion and yields were determined by GC analysis. [b]LDH (105 mg).

Table S5 Effects of TEMPO on disproportionation of 2-cyclohexen-1-one (**4b**)[a]

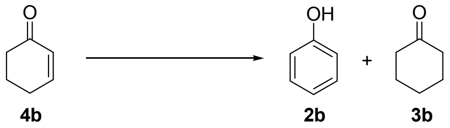

entry	catalyst	additive	conv. (%)	yield (%)	
				**2b**	**3b**
1	Au/LDH	–	27	7	nd
2	Au/LDH	TEMPO	71	41	nd
3	Au_9Pd_1/LDH	–	99	62	19
4	Au_9Pd_1/LDH	TEMPO	99	89	1
5	Pd/LDH	–	99	42	41
6	Pd/LDH	TEMPO	>99	67	16

[a]Reaction conditions: **4b** (0.5 mmol), catalyst (total amount of metals: 3.6 mol%), TEMPO (1 equiv), DMA (2 mL), 130 °C, air (1 atm), 15 min. Conversion and yields were determined by GC analysis.

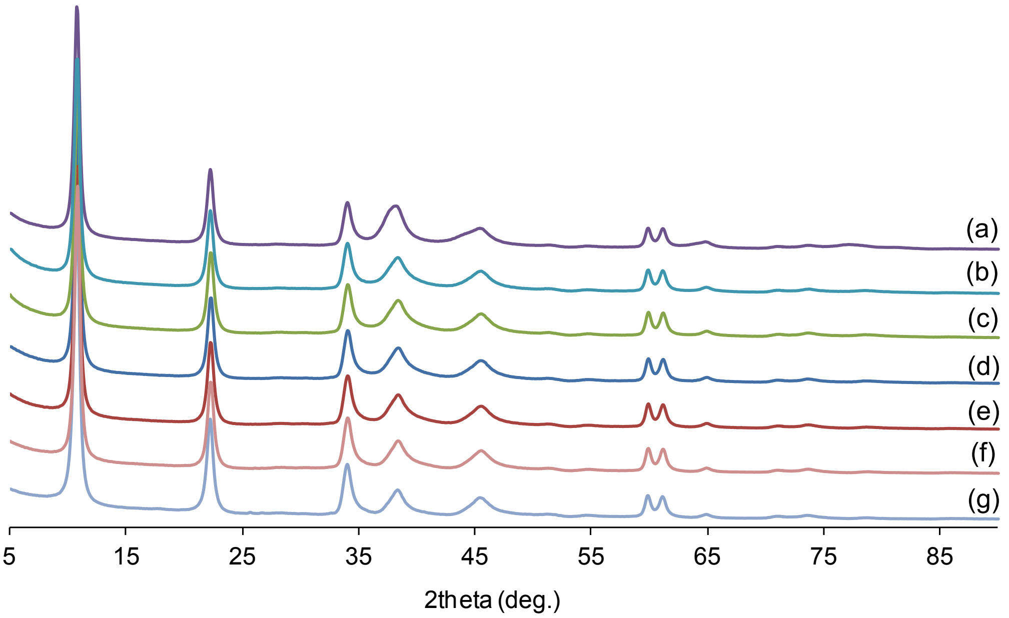

Fig. S1 XRD patterns of (a) Au/LDH, (b) Au_9Pd_1/LDH, (c) Au_4Pd_1/LDH, (d) Au_1Pd_1/LDH, (e) Au_1Pd_4/LDH, (f) Pd/LDH, and (g) LDH.

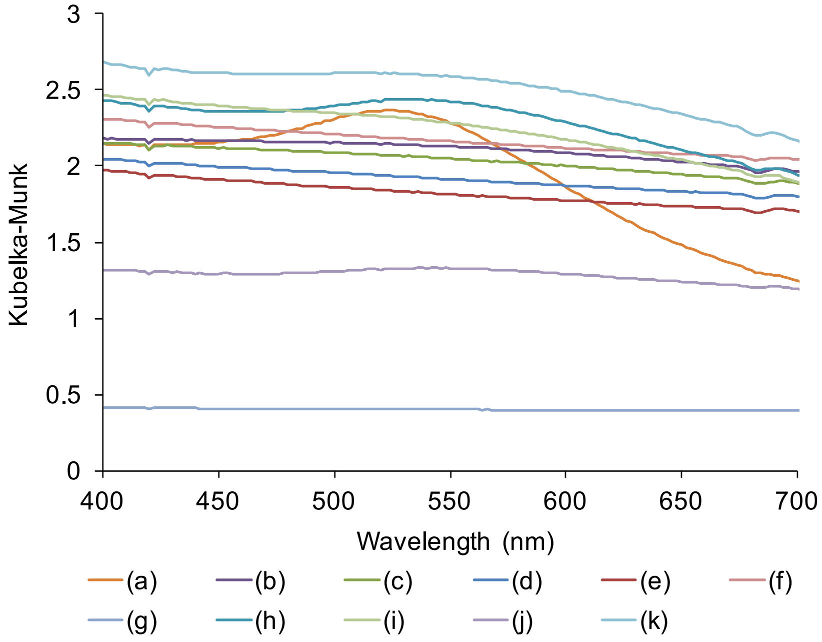

Fig. S2 UV-Vis spectra of (a) Au/LDH, (b) Au_9Pd_1/LDH, (c) Au_4Pd_1/LDH, (d) Au_1Pd_1/LDH, (e) Au_1Pd_4/LDH, (f) Pd/LDH, (g) LDH, (h) Au_9Pd_1/Al_2O_3, (i) Au_9Pd_1/TiO_2, (j) Au_9Pd_1/MgO, and (k) Au_9Pd_1/CeO_2.

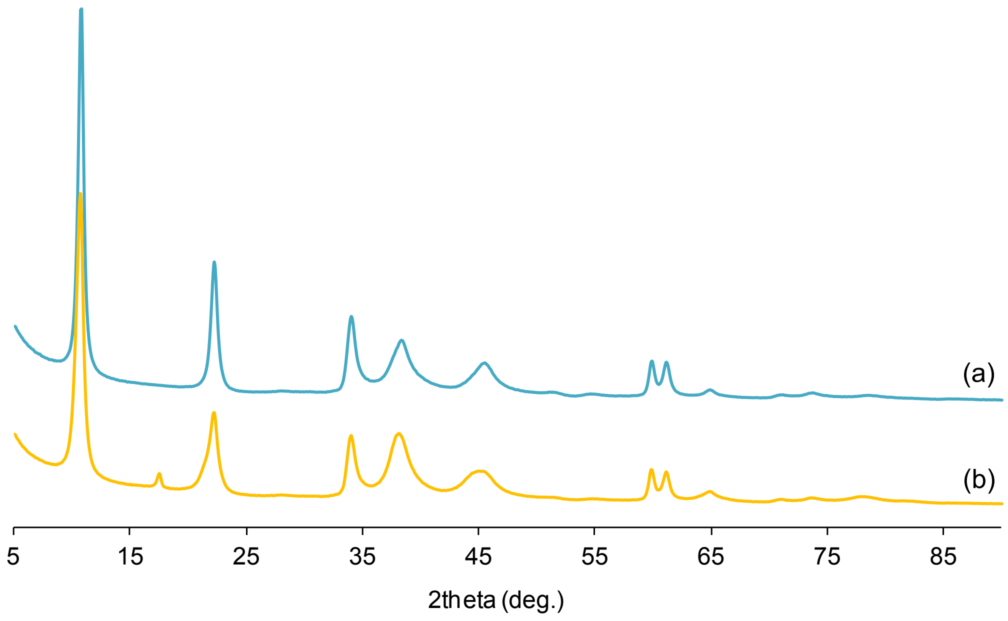

Fig. S3 XRD patterns of (a) fresh Au_9Pd_1/LDH, and (b) Au_9Pd_1/LDH after 10th reuse experiment.

(a)

(b)

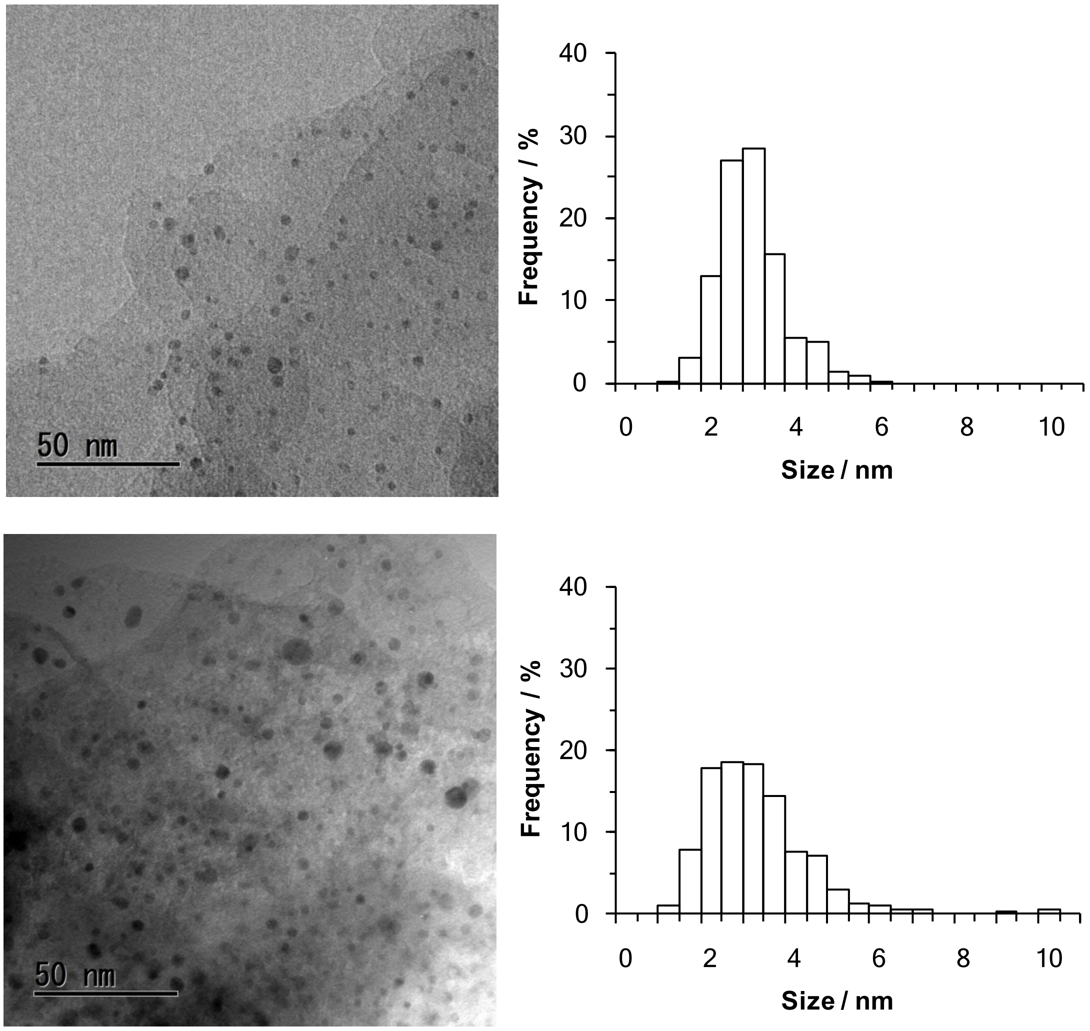

Fig. S4 TEM images and Au–Pd alloy nanoparticle size distributions of (a) fresh Au_9Pd_1/LDH (average: 3.2 nm, σ: 0.8 nm) and (b) Au_9Pd_1/LDH after the 10th reuse experiment (average: 3.3 nm, σ: 1.2 nm). The size distributions were determined using 400 particles.

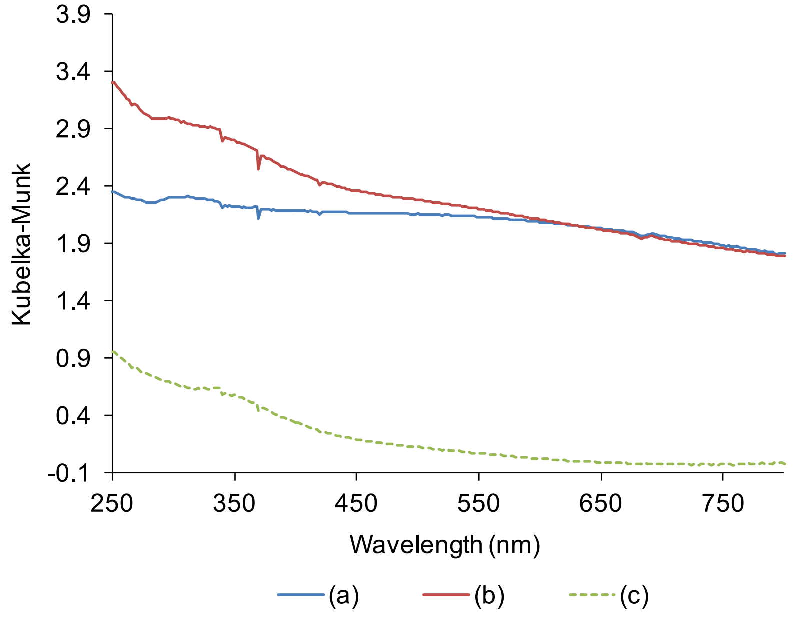

Fig. S5 UV-Vis spectra of (a) fresh Au_9Pd_1/LDH, (b) Au_9Pd_1/LDH after 10th reuse experiment, and (c) the difference spectrum between (a) and (b).

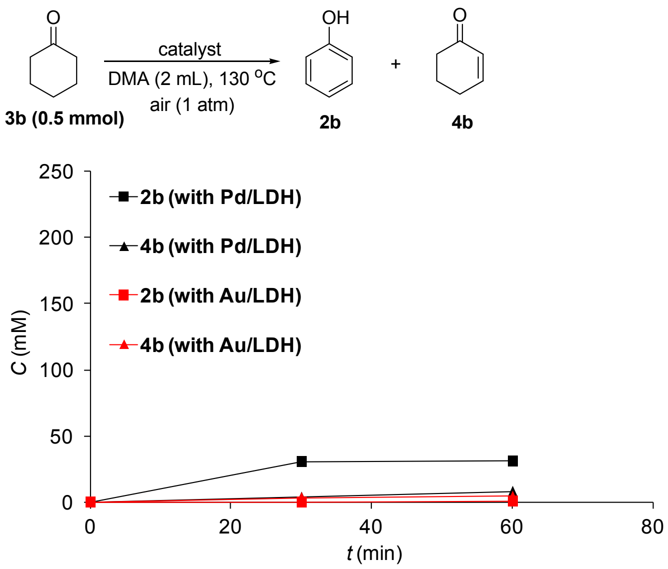

Fig. S6 Dehydrogenative aromatization of **3b** with Pd/LDH or Au/LDH. Reaction conditions: **3b** (0.5 mmol), catalyst (total amount of metals: 3.6 mol%), DMA (2 mL), 130 °C, air (1 atm). GC yields are shown here.

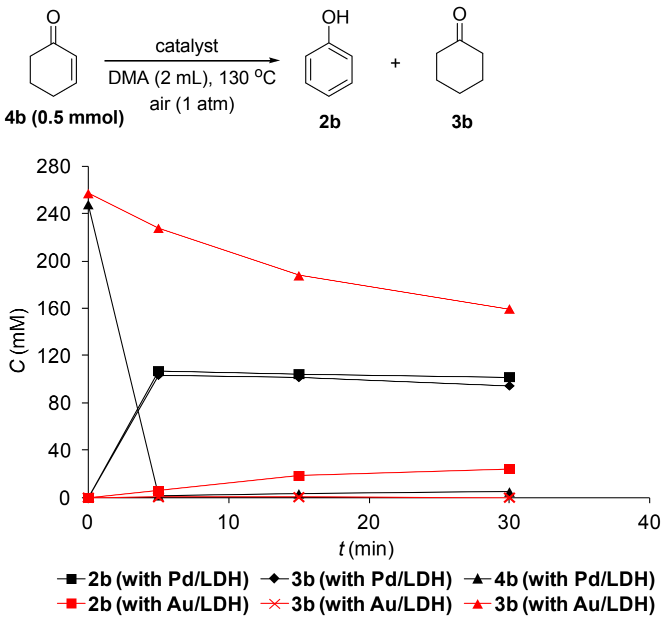

Fig. S7 Disproportionation of **4b** with Pd/LDH or Au/LDH. Reaction conditions: **3b** (0.5 mmol), catalyst (total amount of metals: 3.6 mol%), DMA (2 mL), 130 °C, air (1 atm). GC yields are shown here.

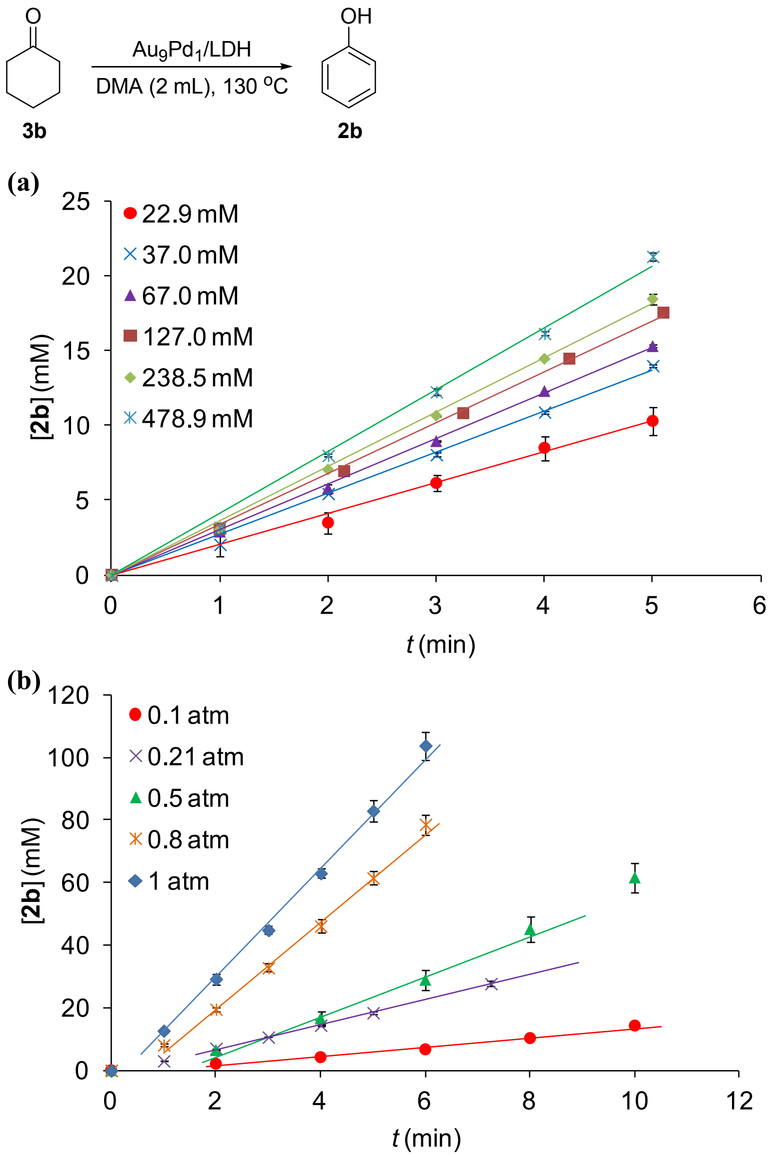

Fig. S8 Reaction profiles for dehydrogenative aromatization of **3b**. Reaction conditions: (a) **3b** (22.9−478.9 mM), Au_9Pd_1/LDH (total amount of metals: 3.6 mol%), DMA (2 mL), 130 °C, air (1 atm); (b) **3b** (250 mM), Au_9Pd_1/LDH (total amount of metals: 3.6 mol%), DMA (2 mL), 130 °C, O_2 (0.1−1 atm). GC yields are shown here.

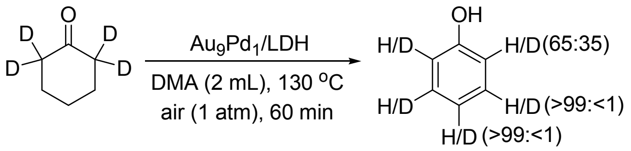

Scheme S1 Oxidative dehydrogenative aromatization of cyclohexanone-2,2,6,6-d_4. Reaction conditions: substrate (0.5 mmol), Au_9Pd_1/LDH (total amount of metals: 3.6 mol%), DMA (2 mL), 130 °C, air (1 atm), 60 min. Conversion and yields were determined by GC analysis.
